# Marketing Strategies in Nanomaterials for Sensor Applications: Bridging Lab to Market

**DOI:** 10.1002/gch2.202400294

**Published:** 2025-03-17

**Authors:** Anoop Singh, Eliyash Ahmed, Mehrajud Din Rather, Atchaya Sundararajan, Alka Sharma, Farah S. Choudhary, Ashok K. Sundramoorthy, Saurav Dixit, Nikolai Ivanovich Vatin, Sandeep Arya

**Affiliations:** ^1^ Department of Physics Govt. Women Degree College Gandhinagar Higher Education Department Jammu Jammu & Kashmir 180004 India; ^2^ Department of Physics (H&S) Guru Nanak Institutions Technical Campus Hyderabad 501506 India; ^3^ Department of Physics University Institute of Engineering and Technology Guru Nanak University Ibrahimpatnam Telangana 501506 India; ^4^ Department of Physics School of Electrical and Electronics Engineering (SEEE) SASTRA Deemed University Thanjavur 613401 India; ^5^ The Business School University of Jammu Jammu Jammu and Kashmir 180006 India; ^6^ Centre for Nano‐Biosensors Department of Prosthodontics Saveetha Institute of Medical and Technical Sciences Saveetha Dental College and Hospitals Poonamallee High Road, Velappanchavadi Chennai Tamil Nadu 600077 India; ^7^ Centre of Research Impact and Outcome Chitkara University Rajpura Punjab 140417 India; ^8^ Peter the Great St Petersburg Polytechnic University Russian Federation St. Petersburg 195251 Russia; ^9^ Division of Research & Innovation Uttaranchal University Dehradun Uttarakhand 248007 India; ^10^ Division of Research and Development Lovely Professional University Phagwara Punjab 144001 India; ^11^ Chitkara Centre for Research and Development Chitkara University Himachal Pradesh 174103 India; ^12^ Department of Physics University of Jammu Jammu Jammu and Kashmir 180006 India

**Keywords:** commercialization process, intellectual property, nanomaterials, sensor, technology transfer

## Abstract

Nanomaterials have revolutionized sensor technology by offering enhanced sensitivity, selectivity, and miniaturization capabilities. However, the commercialization of nanomaterial‐based sensors remains challenging due to the complexities involved in bridging laboratory innovations to market‐ready products. This review article explores the various marketing strategies that can facilitate the successful commercialization of nanomaterials for sensor applications. It emphasizes the importance of understanding market needs, regulatory landscapes, and the value proposition of nanomaterials over traditional materials. The study also highlights the role of strategic partnerships, intellectual property management, and customer education in overcoming market entry barriers. Through a comprehensive analysis of case studies and industry practices, this review provides a framework for companies and researchers to effectively transition from lab‐scale innovations to commercially viable sensor products. The findings suggest that a well‐rounded marketing strategy, combined with robust product development and stakeholder engagement, is crucial for capitalizing on the unique benefits of nanomaterials in sensor applications.

## Graphical abstract

This work discusses strategies for the successful commercialization of nanomaterial‐based sensors, including regulatory compliance and industry collaboration. It highlights unique value propositions, intellectual property protection, and customer education to enhance market acceptance. Future strategies will evolve with technological advancements, sustainability trends, and consumer demands for seamless market integration.

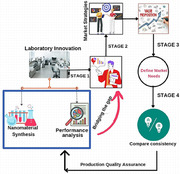

## 1 Introduction

Scientific and technical advancements have been greatly aided by the nanoworld. The area of nanotechnology underwent a revolution with the discovery of nanomaterials including carbon fullerenes, carbon nanotubes, and structured mesoporous materials in 1985, 1991, and 1992.^[^ ^1^ ^]^ Based on their dimensionality, nanomaterials may be classified into a number of categories, including one‐dimensional (nanotube, nanowire), two‐dimensional (nanofilms), and three‐dimensional (nanoparticles), among others. Since nanomaterials exhibit distinctive mechanical, electrical, magnetic, and optical characteristics, nanomaterials are among the materials that are investigated the most. These emerging features have grabbed consumers' interest in various applications like biomedical, optoelectronic devices, batteries, gas sensors, catalysts, and agricultural applications.^[^ ^2^, ^3^ ^]^ Particular types of nanomaterials, such as mixed and pure oxides and organic‐based materials, have drawn increasing interest in recent years for gas sensing applications due to their remarkable ability to improve sensitivity, selectivity, and reaction time. Wide band gap nanomaterials have been shown to be superior gas‐sensing materials with high responsiveness. Since most carriers are confined in the surface state and nanosized grains have less conductivity than bulk under ambient circumstances, it makes sense that nanomaterials‐based gas sensors have an advantage over bulk ones. Therefore, compared to bulk‐sized grain, they show larger conductance changes when exposed to gas because more carriers are activated from their trapped states to the conduction band. It is well established that determining a nanomaterial's morphologies, particle sizes, and dimensions is crucial to understanding its sensing properties.^[^ ^4^ ^]^ Among them, the gas sensitivity is influenced by the crystallite size; that is, the maximal sensitivity is only attained if the film's crystallite size and space charge layer thickness are equivalent.^[^ ^5^, ^6^, ^7^ ^]^ Differently shaped nanomaterials, such as size confinement in two dimensions, also provide improved sensitivity to surface chemical reactions because of their huge surface‐to‐volume ratio and narrow diameters that are equivalent to Debye lengths.^[^ ^8^, ^9^, ^10^, ^11^, ^12^ ^]^ To accomplish the aforementioned, there are several ways. Generally speaking, there are two categories of pure nanomaterials: p‐type and n‐type nanomaterials. Many nanomaterials have so far been effectively utilized as materials for sensing to identify oxidizing and reducing gases by converting information into signals (electrical) when exposed to the appropriate test gas.^[^ ^13^, ^14^, ^15^, ^16^, ^17^ ^]^ Two methods can be used to synthesize nanomaterials: the bottom‐up approach (solvothermal, sol–gel, electrochemical method, chemical method, etc.), which builds the material atom by atom, and the top‐down method (lithography, laser ablation, ball milling,), which starts with bulk materials and synthesizes the material from there.^[^ ^18^, ^19^, ^20^, ^21^, ^22^ ^]^ Numerous researchers have shown, according to a review of the literature, that numerous types of nanomaterials, such as different dopants, catalysts, adhesives, binders, and surfactants, have all been employed to improve the detecting properties of sensors manufactured from these materials. Their film production process offers an additional option for sensor design in addition to the previously mentioned ones.^[^ ^23^, ^24^, ^25^ ^]^

**Figure 1:**
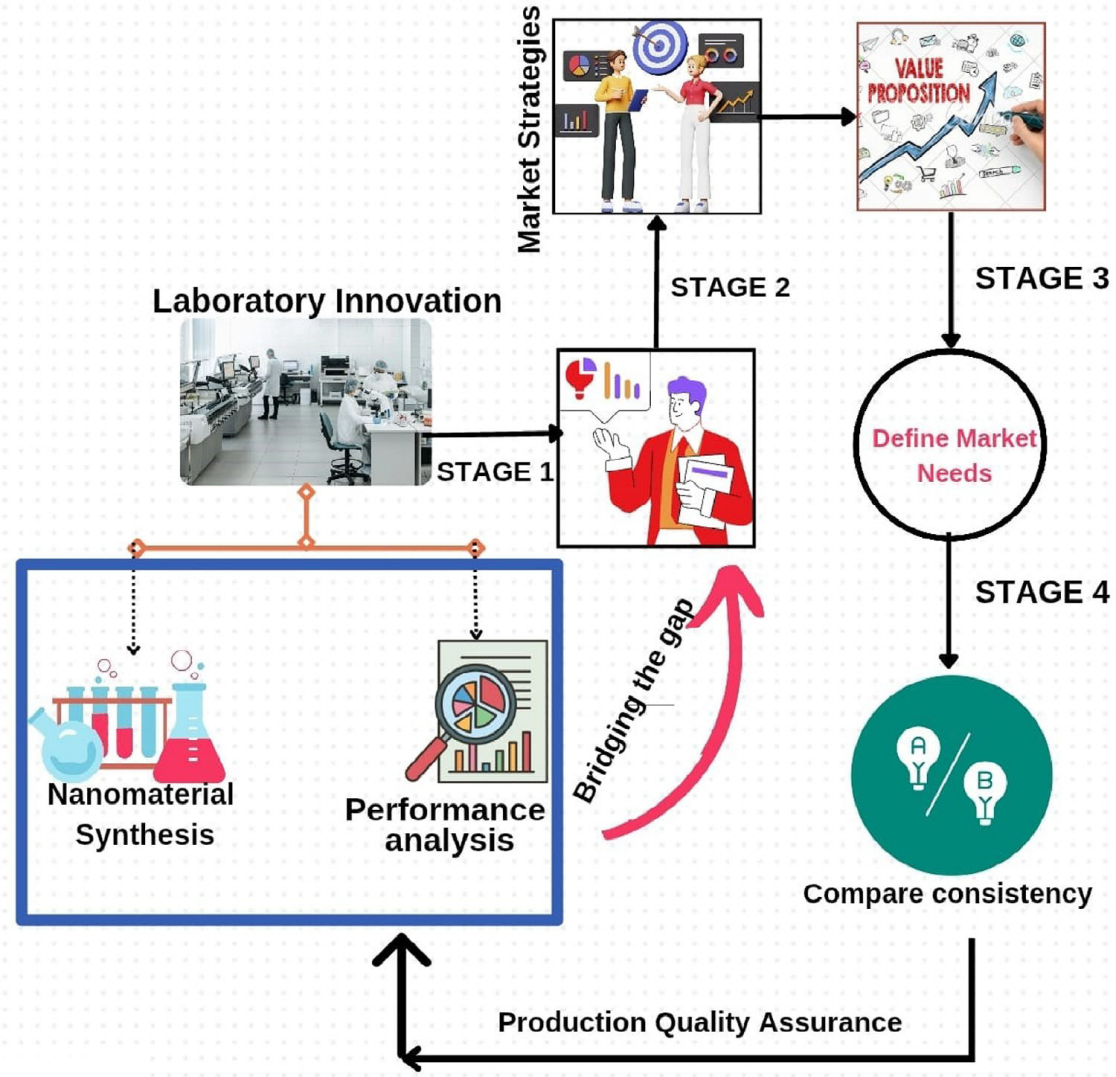
The overall novelty of review.

This article will provide a brief overview of nanomaterials for sensing applications, concentrating on the use of various nanomaterials for gas sensors, their film deposition technique, and their basic principles. The field of sensor technology is undergoing a rapid transformation, driven by the unique properties of nanomaterials. These materials, with their exceptional surface area, quantum confinement effects, and tunable properties, offer the potential to create sensors with unprecedented sensitivity, selectivity, and versatility. This is reflected in the growing market for nanomaterial‐based sensors. According to Grand View Research,^[^ ^26^ ^]^ the global nanomaterial‐based sensor market was valued at ≈$12.42 billion in 2023, and is projected to reach $32.77 billion by 2030, exhibiting a robust compound annual growth rate (CAGR) of 15% during the forecast period (2023–2030). This growth is fueled by increasing demand for advanced sensors in various sectors, including healthcare, environmental monitoring, aerospace, defense, and consumer electronics. The overall novelty of the review article is demonstrated in **Figure** **1**. The several variables that directly affect the selectivity, sensitivity, and response time of nanomaterials will also be covered in this review article.

## 2 Nanomaterials in Sensor Development

### 2.1 Carbon‐Based Nanomaterials

Modern life is increasingly reliant on nanosensors, especially in the healthcare industry where point‐of‐care gadgets, personalized treatment, and more affordable and accurate diagnostic tools are needed. Since carbon nanomaterials have such a broad potential window, quick electron transfer kinetics, low residual current, fluorescent characteristics, and easily renewable surfaces, they have attracted researchers' attention as promising transduction materials.^[^ ^27^ ^]^ Carbon nanomaterials have been the subject of extensive research efforts aimed at creating extremely selective and sensitive nanosensors. Biological and chemical analytes, such as mycotoxins, pantothenic acid, protein, folic acid, airborne bacteria, as well as ascorbic acid, uric acid, and norepinephrine, have all been the subject of intense research interest in recent years due to the sensitive detection capabilities of nanosensor platforms. Compared to conventional materials, the utilization of carbon nanomaterials in nanosensor platforms offers a number of benefits, including high biocompatibility, fast electron transfer kinetics, improved interfacial adsorption properties, and good electrocatalytic activity. Several methods exist for integrating these nanoparticles into electrochemical sensors, including polymer‐based coatings,^[^ ^30^ ^]^ direct growth on a substrate,^[^ ^29^ ^]^ drop casting,^[^ ^28^ ^]^ utilization of binders like dihexadecyl hydrogen phosphate or Nafion,^[^ ^31^ ^]^ and screen printing.^[^ ^32^ ^]^ The direct growth of carbon nanomaterials over the electrode surface produces a more uniform coating and facilitates the batch manufacture of nanosensors as compared to drop casting or dip coating.^[^ ^33^ ^]^ Moreover, polymer‐based coatings may support the dispersion of carbon nanomaterials for deposition as well as their chemical and physical characteristics. At times, it is desirable to include more metallic nanoparticles in the polymer matrix in order to maintain the necessary degree of electrode conductivity.^[^ ^34^ ^]^ The main problem nowadays arises due to the accumulation of heavy metal ions. Water bodies' concentration of heavy metal ions directly affects the health of living organisms.^[^ ^35^ ^]^ Several techniques, including atomic absorption spectroscopy (AAS),^[^ ^36^ ^]^ atomic emission spectroscopy,^[^ ^37^ ^]^ inductively coupled plasma mass spectrometry (ICP‐MS),^[^ ^38^ ^]^ and X‐ray fluorescence spectrometry,^[^ ^39^ ^]^ constitute the foundation of standard methods for the measurement of heavy metal contamination. These methods are quite expensive and unsuitable for on‐site analysis. Furthermore, it is impossible to analyze the bio available amounts that are reachable by living things using these methods; they can only measure the overall quantity of heavy metals. One of the most straightforward, sensitive, and accurate techniques for identifying metal contamination in food and the environment is electrochemical detection utilizing nanosensors. Numerous advantages are provided by electrochemical sensing methods, including cheap cost, mobility, high sensitivity, quick analysis times, and simple flexibility for in situ detections.^[^ ^40^ ^]^ To increase the sensitivity of the nanosensors, nanomaterials may be added to the three‐electrode electrochemical system's working electrode.^[^ ^41^ ^]^ Upon identification of an analyte, these nanosensors are then used by monitoring changes in their electroluminescence, electrochemical impedance, current, and potential.^[^ ^42^ ^]^ When it comes to materials utilized as adsorbents, preconcentrator agents, or transducers in the creation of nanosensors, nanomaterials based on carbon are the most intriguing. Analytes that are both organic and inorganic may be detected by the carbon nanomaterials. Highly sensitive and selective metal ion sensing is made possible by functionalizing nanomaterials of carbon with biological recognition components (such as DNA, antibodies, enzymes, or microbes).^[^ ^43^ ^]^ In particular, because of their stability in biological pH settings, DNA‐based nanosensors have attracted a lot of scientific attention lately for the detection of heavy metal ions.^[^ ^44^ ^]^ According to Wen et al.,^[^ ^45^ ^]^ graphene oxide/Prussian blue nanoparticles modified with DNA may be used to detect arsenite. The food business is seeing a rapid increase in innovation concerning the creation and use of food additives, pesticides, and materials for food processing, coating, and packaging. In agriculture, pesticides are heavily used to increase yields by managing weeds, insects, and other pests. To increase food safety and shelf life, functional characteristics are added to food. Unwantedly high levels of food additives and unintentional pesticide and veterinary medicine contamination in food and water sources have grown to be serious health concerns. The residues left behind by pesticides are very toxic and may cause cholinergic dysfunction and other health problems in both people and animals. Food samples may be contaminated by pesticides, veterinary drug residues, or toxic food additives. Toxicological contaminations are typically analyzed using techniques like capillary electrophoresis,^[^ ^49^ ^]^ gas chromatography‐mass spectrometry,^[^ ^48^ ^]^ high‐performance liquid chromatography (HPLC),^[^ ^46^ ^]^ and HPLC‐mass spectrometry.^[^ ^47^ ^]^ The electrochemical biosensing of pollutants in food and water has been described as the basis for the creation of nanosensors. The electrochemical biosensors function quickly and have excellent repeatability, sensitivity, and selectivity.^[^ ^50^, ^51^ ^]^ Carbon nanostructures are included to provide excellent stability and to improve the loading of bioreceptors on the electrode surface. Additionally, carbon nanoparticles act as a relay to transmit electrons from biomolecules to the electrode. A good limit of detection (0.003 mm) for the specific identification of Sudan I (a coloring agent) was reported by Elyasi et al.^[^ ^52^ ^]^ using an ionic liquid carbon electrode modified with a Pt/CNT nanocomposite.

### 2.2 Noble Metal Nanoparticles

**Figure 2:**
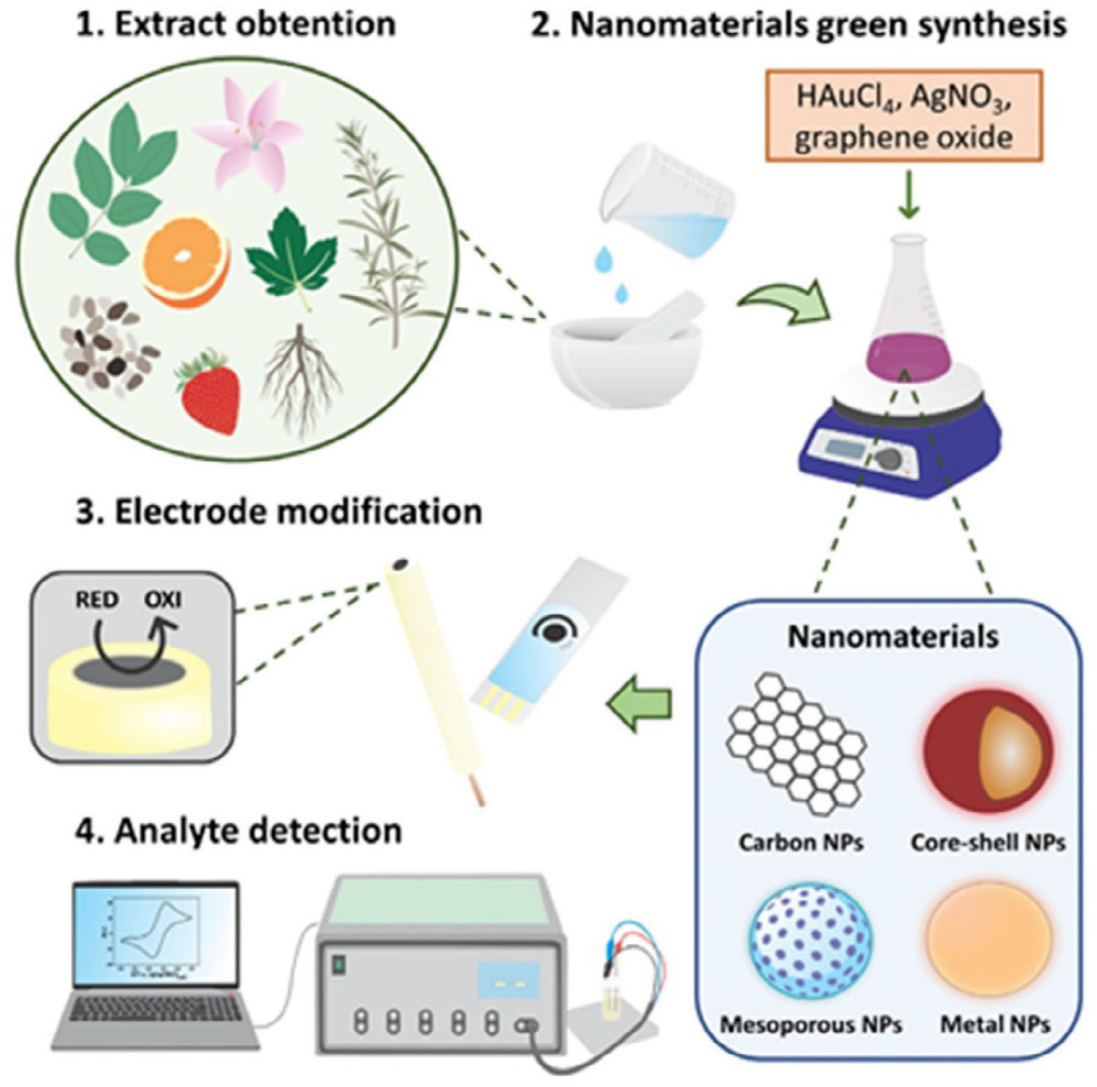
An outline of how to biosynthesize nanoparticles from vegetal materials and immobilize them in electrochemical transducers for use in sensors. Reproduced with permission, Copyright 2022, Elsevier.^[^ ^55^ ^]^

Noble metal nanoparticles (NPs) such as gold (Au) and Silver (Ag) offer great structures for building sensing devices owing to their high S/V (surface‐to‐volume) ratio. Their excellent electrical and optical features are particularly sensitive to changes in environment. These qualities guarantee that sensory mechanisms are very sensitive. **Figure** **2** displays a thorough schematic illustrating the method of nanomaterials biosynthesizing utilizing samples based on plants and then immobilizing them in transducers (electrochemical) for diverse uses in sensing. This entire strategy is distinguished by its cost‐effectiveness, simplicity, and its ability to dramatically boost sensor fabrication operations and detecting abilities. Colorimetric sensing approaches that employ nanomaterials primarily depend on variations in their optical characteristics owing to changes in shape, dispersion, and aggregation. These alterations may result in shifts of wavelength (plasmon resonance), which can be monitored to discriminate between diverse analytes.^[^ ^53^, ^54^ ^]^ The utilization of ecologically benign materials, notably extracts of plant, for the manufacture of MNPs revealed various advantages in medicinal and other biological uses. The GS approach was also extremely economical and may be employed as an appropriate substitute for the large‐scale manufacture of MNPs. Colorimetric sensors based on AgNP operate by sensing variations in absorption (optical) when come into touch with certain substances. This progressive variation in optical characteristics may be exploited to develop a real sensor (optical) that senses the particular level of analyte. The sensitivity of the developed sensors relies on the functional group responsible for modifying the characteristics of the AgNPs, which in turn modifies the measured band shape, energy, and intensity of surface plasmon resonance (SPR).

Unmodified, green‐synthesized AgNPs serve as Hg^2+^ detection colorimetric sensors in a technique developed by Farhadi et al to detect Hg^2+^ in aqueous environmental samples. This redox interaction between AgNPs and Hg^2+^ in the solution drives the AgNPs‐Hg^2+^ reaction.^[^ ^56^ ^]^ Initially, they devised a simple and low‐cost technique for creating stable AgNPs using a bio‐reduction procedure. For this, aqueous extract (manna of Hedysarum) was used as the reducing agent in a solution of AgNO_3_, and soap‐root extract was used as the stabilizer. The yellow‐brown AgNP fresh solution was readily visible to the unaided eye to become colorless when Hg^2+^ was present, along with the SPR band's blue shifting and widening. With 2.2 × 10^−6^ m limit of detection, this approach has been verified with several ions of metal and has shown to be extremely selective for Hg^2+^ over other metals.^[^ ^57^ ^]^ While salts of ammonium are used as additives in food and cleaning agents, ammonia is a versatile substance that finds use in a variety of industries, including fertilizer, animal feed, pharmaceutical, paper, plastic, fiber, and explosives. It is observed that salts of ammonia are caustic and hazardous, posing high health risks to crustaceans, fish, and humans. Particularly aquatic animals are vulnerable to harmful effects at high concentrations because they excrete ammonia and are unable to transform it into less dangerous molecules. As a result, it is essential to identify, feel, and keep an eye on water's ammonia levels. Through the GS of AgNPs, sensors (optical)for the sensing of ammonia (dissolved)were created using sugarcane leaf extract,^[^ ^60^ ^]^ guar gum (polysaccharide *Cyamopsis tetragona* loba),^[^ ^59^ ^]^ and aqueous fruit extract of *Terminalia chebula*.^[^ ^58^ ^]^ AgNP production was seen by color changes in solutions containing ammonia, indicating the creation of complex (ammonia‐AgNP) that increased and changed the intensity of absorbance (SPR). 1 ppm detection limit was found for the solution of ammonia when the polysaccharide Cyamopsis tetragona loba was used. Silver nanoparticles produced by green synthesis are capable of detecting additional dangerous heavy metal ions in water, including Cu^4+^, Cd^2+^, and Cr^3+^. Human life and health are at risk due to these harmful elements. Based on AgNPs stabilized lignin from Acacia wood, a comprehensive colorimetric sensor was created to assess the detection of various heavy metal ions throughout the wide range of 100 mm to 1 nm.^[^ ^61^ ^]^ AgNP's reduction of metal ions and the successive deposition of metal on the nanoparticles' surface cause a change in the absorption peak. Additionally, AgNPs were developed to identify cadmium, chromium, and copper ions in water by using a variety of plant extracts. In particular, AgNPs with a 0.249 mm limit of detection were created utilizing Moringa oleifera flower extract to identify copper ions.^[^ ^62^ ^]^ Without any surface functionalization, researchers created AgNP by detecting cadmium ions using an extract from Allium sativum.^[^ ^63^ ^]^ They determined the detection limit of the device to be 0.277 µm using DPV. AgNPs from Lycopersicon esculentum extract are used by another group to create a novel kind of sensor that detects Cr ions.^[^ ^64^ ^]^ The DPV findings for this system indicated a linear calibration range of 10–90 µm, with a detection limit of 0.804 µm. Numerous studies have described electrochemical sensors that use green‐synthesized AgNPs on modified GC electrodes to identify target biomolecules. Recently, AgNPs were produced in an environmentally friendly way using pollen extract from *C. sempervirens*. After the obtained AgNPs were modified to create AgNP–GCE, a GC electrode was tested on H_2_O_2_ and a 0.23 µm limit of detection was obtained.^[^ ^65^ ^]^ In previous investigations, a GC improved with bio‐hybrids of AgNPs, generated utilizing pine nuts Araucaria angustifolia and nanoplatelets of exfoliated graphite, was applied for the sensing of paracetamol with LOD of 8.50 × 10^−8^ m.^[^ ^66^ ^]^

**Figure 3:**
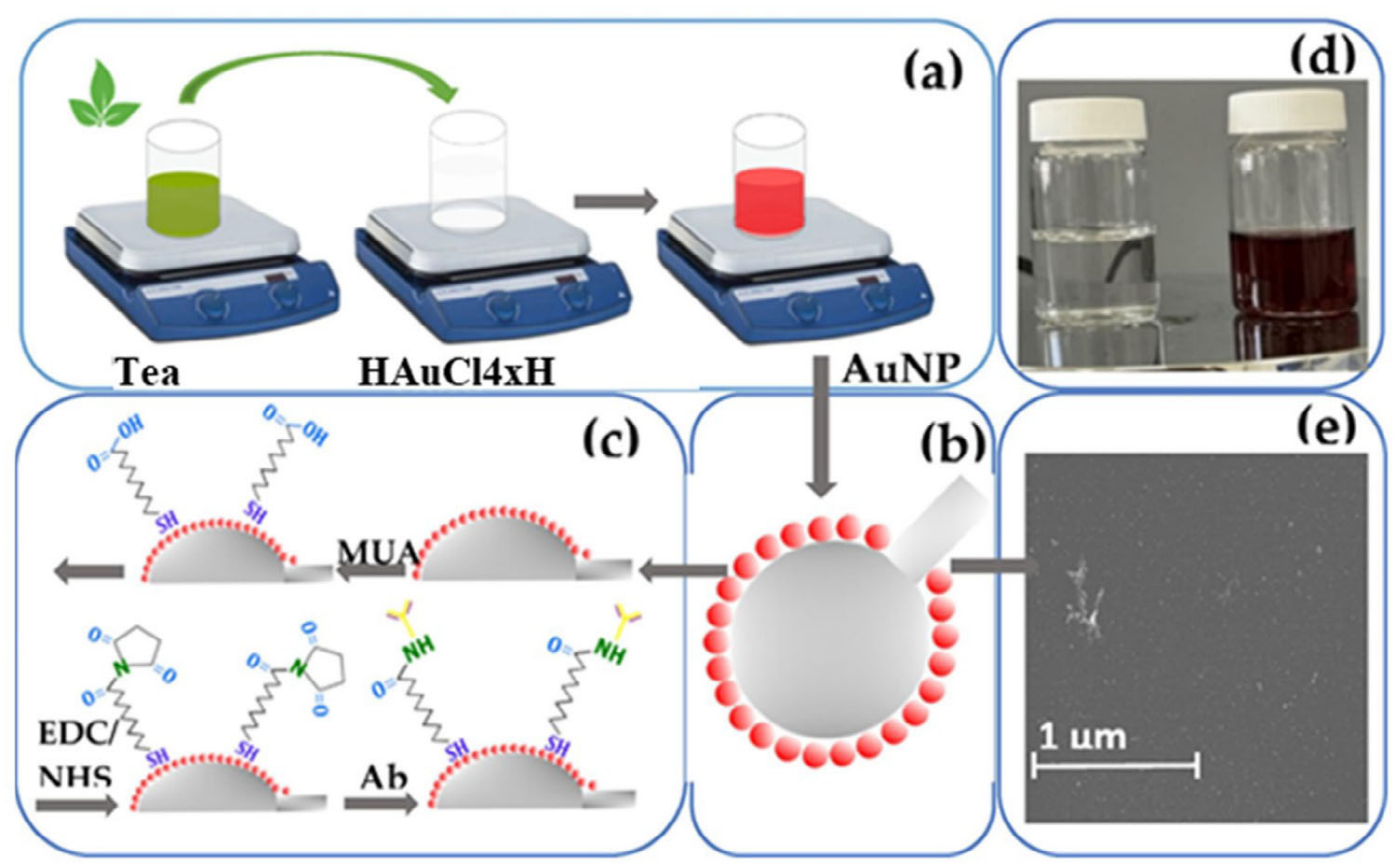
Diagram illustrating the complete functionalization of ball resonator optical fibers with green‐synthesized AuNPs embellished with biological recognition components for analyte detection: a) the process of creating AuNPs using green tea extract; b) the APTMS treatment causing AuNPs to become immobile on the ball resonator optical fiber surface; c) the antibody‐mediated functionalization of the gold‐coated sensor after pre‐treatment with MUA and EDC/NHS; d) the visible change of color during synthesis from colorless to wine‐red; e) the SEM picture showing the surface of the optical fibers linked to AuNPs in a ball resonator. Reproduced with permission, Copyright 2023, Elsevier.^[^ ^72^ ^]^

The prospective applications of gold nanoparticles (AuNPs) in fields including electrochemistry, catalysis, and optical sensors are driving up their popularity. Using GS techniques to extract these NPs from plants offers several benefits over conventional chemical and physical techniques. Applied to different biological assemblies or alterations, AuNPs' numerous surface functions make them very flexible and adaptive, leading to enhanced applications. High pressure, energy‐intensive settings, high temperatures, or hazardous chemicals are not necessary for this environmentally benign method, which is also readily scalable. Because plants are easily obtained and safe to work with, this synthesis approach seems promise in comparison to other methods. The fact that this process is generally accessible, safe to use, and may be developed using ordinary concepts are other advantages.^[^ ^67^, ^68^ ^]^ Guar gum (GG) has been used as a reducing agent to manufacture AuNPs via GS, and this has led to the creation of sensor (optical) that can sense ammonia in aqueous form.^[^ ^69^ ^]^ This technique has shown remarkable reproducibility, with reaction times of around 10 s and remarkable sensitivity, capable of identifying very low concentrations, i.e., 1 ppb. Green AuNPs were produced by using a different polysaccharide (plant‐derived) from Gum Karaya as a reducing, stabilizing, and functionalizing agent. When these nanoparticles come into contact with copper ions, they shift from red to blue, which makes them ideal for detecting copper in biological and actual water samples. With a detection limit of 10 nm, the detection system showed a significant linear correlation (*R* ^2^ = 0.998) over a linear range of 10 to 1000 nm of Cu^2+^.^[^ ^70^ ^]^ Cysteine is a light aminothiol that may be detected in human plasma using a colorimetric sensor.^[^ ^71^ ^]^ The AuNPs from the willow tree bark extract served as the basis for this sensor. The outcome of the interaction between the cysteine and synthesized nanoparticles was a purple color shift in the SPR band. Within a certain range, the intensity of the color shift correlated with the cysteine content. As shown in **Figure** **3**, a recent work has developed a biosensing platform for identifying the CD44 cancer biomarker utilizing AuNPs produced by green tea leaf synthesis.^[^ ^72^ ^]^

Surface of SPE modified with a biohybrid comprising AuNPs/rGO assembly synthesis by utilizing *E. tereticornis* leaves as the sustainable reducing agent to detect L‐tryptophan (Trp) amount in samples (biological).^[^ ^73^ ^]^ In a range from 500 and 0.5 m, the best circumstances resulted in LOD and LOQ values of 0.39 and 1.32 µm, respectively. Another study that used rose water as a reducing agent to address glucose detection at rGO/AuNPs reported a 10 µm LOD for 1–8 mm linear range.^[^ ^74^, ^75^ ^]^ Additionally, the sensor demonstrated long‐term storage stability, anti‐interference capability, repeatability, and reproducibility. Another method involved modifying a carbon nanotube screen‐printed electrode by dropping‐casting the AuNPs from Sargassum. This resulted in the creation of a unique, portable electrochemical sensing platform that could detect glucose at concentrations as low as 50 µm.^[^ ^76^ ^]^

### 2.3 Metal Oxide Nanomaterials

**Figure 4:**
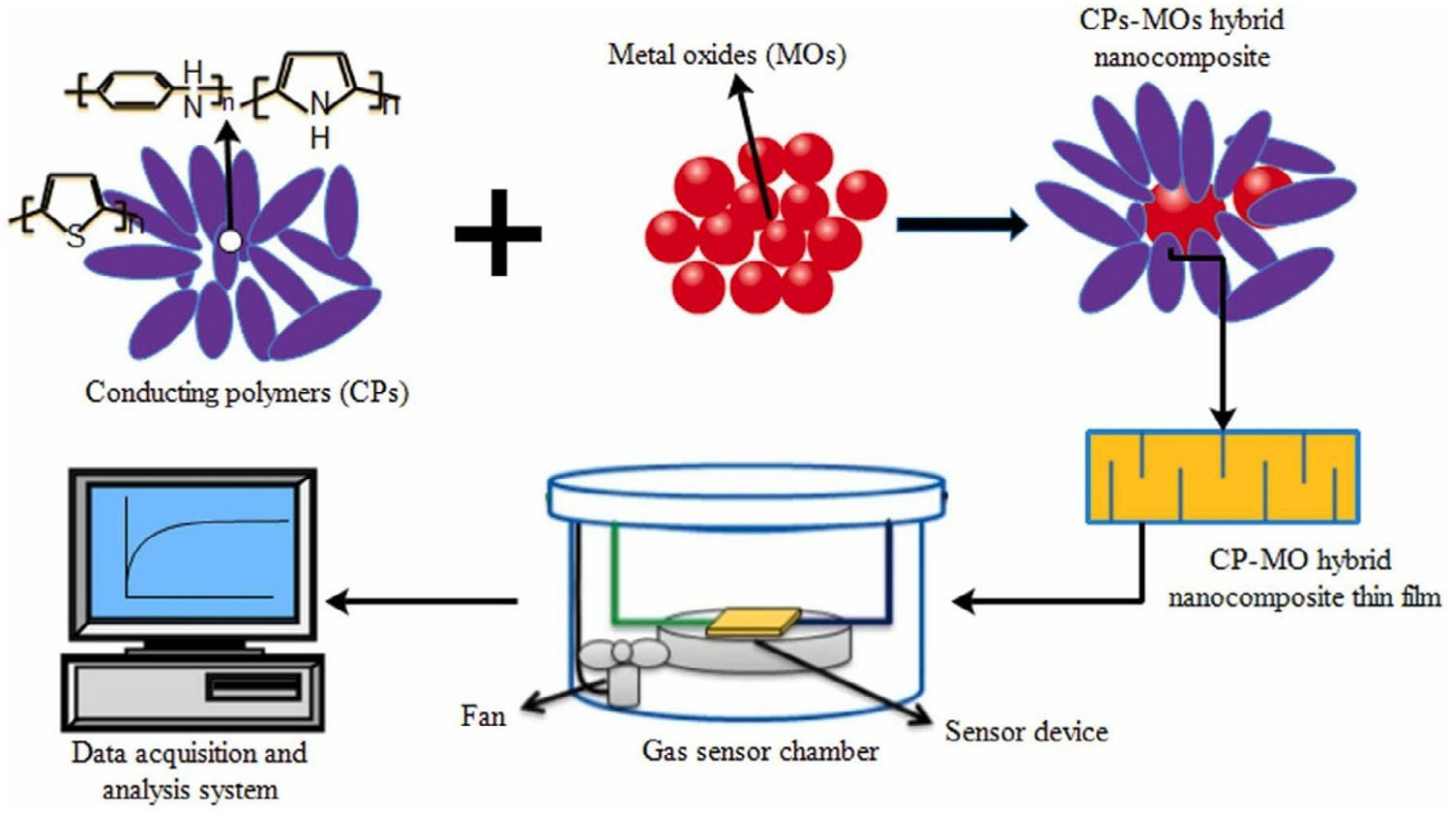
Shows the scheme for preparation of thin films for sensor applications. Reproduced with permission, Copyright 2023, Elsevier.^[^ ^80^ ^]^

We first outline several typical MONM characteristics that may affect how they interact with biomolecules before going into the sensor work.^[^ ^77^ ^]^ We talk about how different anions and tiny molecules combine, how enzyme‐like catalytic activity occurs, and how ligand interaction causes MONMs to dissolve. Water is hydrated via chemisorption, which has the potential to split into surface hydroxide.^[^ ^78^ ^]^ Charged surfaces may be produced by further pronating or deprotonating the surface hydroxide, depending on the pH. Generally speaking, increasing pH promotes surface hydroxide deprotonation, which results in more negatively charged surfaces; but, in acidic buffers, surface hydroxide may be protonated to become positively charged. The point‐of‐zero‐charge (PZC), or pH at which the total charge is zero, is the pH at which a positive potential change to a negative potential. It should be mentioned that the PZC of the same material might vary by a few pH units depending on the preparation technique, particle size, surface hydroxide concentration, characterization technique, and buffer conditions.^[^ ^78^ ^]^ Different MONMs have varying surface charges at a given pH. For example, with just a few exceptions (ZnO, NiO), the majority of oxides are negatively charged at physiological pH. It is unavoidable for MONMs to interact with charged biomolecules, and these electrostatic interactions often have a crucial function in these systems.^[^ ^79^ ^]^ The surface metal species on MONMs may interact with ligands via coordination in addition to electrostatic interactions. The hard‐soft‐acid‐base hypothesis is a helpful place to start in this respect. Hard or borderline metals are often those that have the ability to create stable oxides. Certain metals, like Co^2+^ and Ni^2+^, have sluggish ligand exchange kinetics and strong coordination interactions, which may cause them to bind to their ligands extremely firmly. For sensor applications, the thin films of the synthesized nanomaterials of metal oxide or conducting polymer are required.^[^ ^80^ ^]^ The synthesis procedure for the development of thin films of the nanocomposites of conducting polymer/metal oxide is shown in **Figure** **4**.

**Figure 5:**
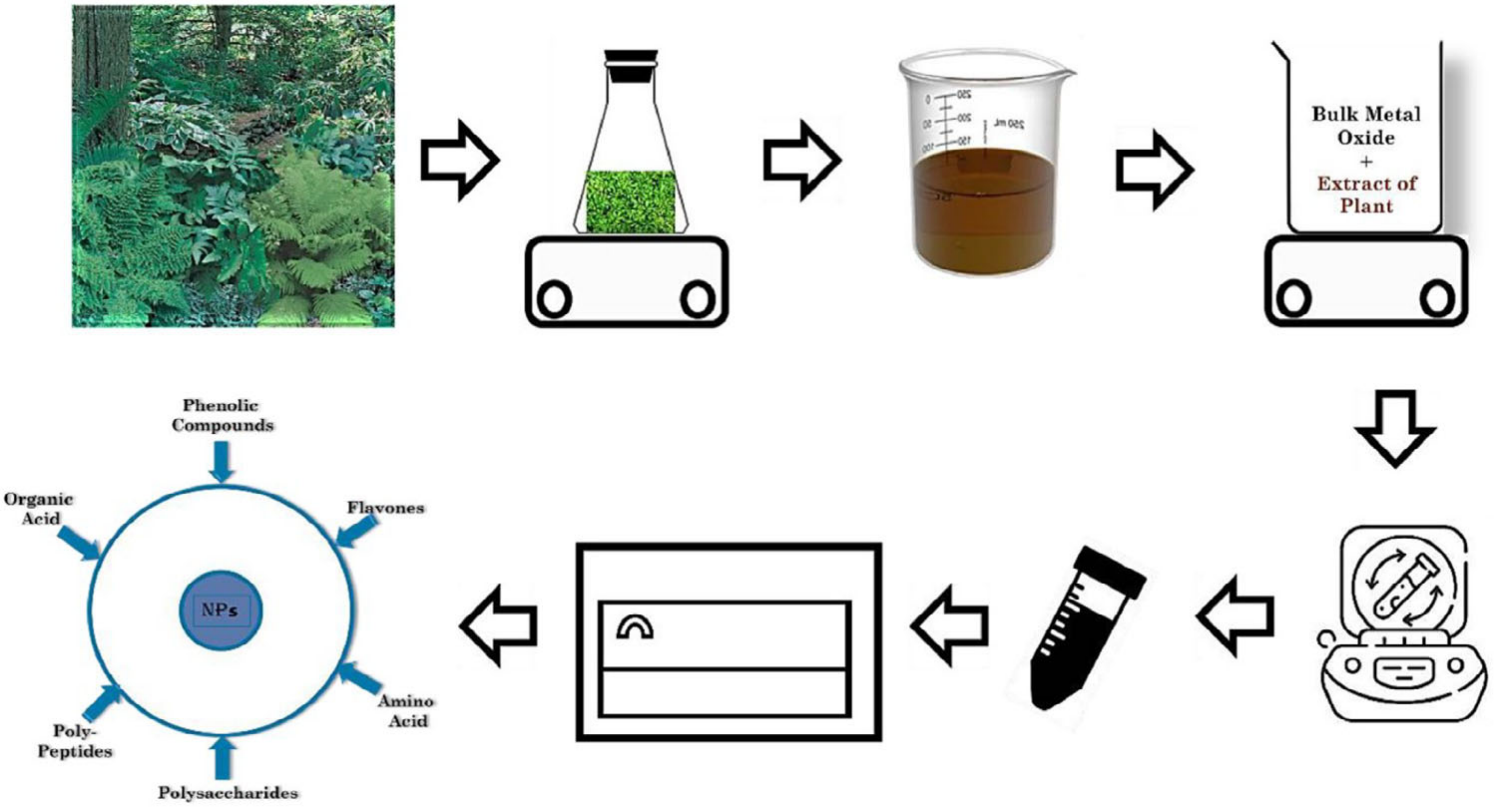
Shows the steps involved in the synthesis of MONM using plant extracts. Reproduced with permission, Copyright 2024, Elsevier.^[^ ^103^ ^]^

While a large number of soft metals may also produce oxides, such as CuO, HgO, Ag_2_O, and CdO, there aren't many uses for them in sensing. These metals often combine with sulfur to generate more stable compounds. These soft thiophilic metals' toxicity is one issue that restricts their use. Inorganic anions are one class of fascinating ligands. For example, the use of magnetic iron oxides to remove arsenate has been researched for decades.^[^ ^81^ ^]^ Although two additional mechanisms are also recognized, arsenate mostly forms a bidentate binuclear complex with surface iron.^[^ ^82^, ^83^ ^]^ There is ample evidence supporting the chemisorption of sulphate, phosphate, halides, nitrate, carbonate, selenate, and oxalate.^[^ ^78^ ^]^ In addition to anions, a few tiny molecules of biological significance may also form strong bonds with the surface metal sites. Dopamine^[^ ^84^ ^]^ and catechols,^[^ ^85^ ^]^ for instance, have a strong affinity for practically all MONMs. These tiny molecules may also act as a bridge to secure DNA and other functional biomolecules on the surface of oxides.^[^ ^86^, ^87^ ^]^ Finally, a variety of contact forces, such as electrostatic, coordination, and hydrogen bonding, may be involved in the adsorption of biomacromolecules on MONMs. We go over some basic theories on DNA adsorption on MONMs in a subsequent section. Perry et al studied how certain common MONMs interact with other biomolecules.^[^ ^79^ ^]^ Even in biological settings, MONMs are often thought to be very stable. Nevertheless, their solubility and ligands may cause them to dissolve. Subsequently, WO_3_, NiO, Sb_2_O_3_, and CoO dissolved considerably in the cell culture media after ZnO and CuO. In terms of metal leaching, other oxides are rather stable. Nonetheless, reducing pH and/or interacting with ligands might hasten dissolution.^[^ ^88^ ^]^ Dopamine, for instance, may interact as a ligand with iron oxide to cause Fe leakage and the formation of an alternative surface complex.^[^ ^89^ ^]^ Glutathione and other biological thiols, such as MnO_2_ sheets, may dissolve and be reduced to free Mn^2+^.^[^ ^90^ ^]^ These MONMs may be harmful due to this dissolution and leaching of metal ions, yet controlled dissolution may also be advantageous for the development of sensors. MONMs feature a variety of significant characteristics that are helpful for the creation of biosensors, including optical (ZnO) and magnetic (Fe_3_O_4_) capabilities. Many additional MONMs were discovered to imitate peroxidase after this groundbreaking study, including Fe_2_O_3_,^[^ ^91^ ^]^ V_2_O_5_,^[^ ^92^ ^]^ Co_3_O_4_,^[^ ^93^, ^94^ ^]^ and CuO.^[^ ^95^ ^]^ Oxidase's capacity to oxidize substrates utilizing dissolved oxygen when H_2_O_2_ is not present is advantageous for analysis. According to a 2009 study by Asati et al.,^[^ ^96^ ^]^ nanoceria may catalyze the oxidation of dopamine, ABTS, and TMB. Though NiO is also an oxidase mimic, it only produces fluorescent resorufan when it combines with Amplex Red (AR).^[^ ^97^ ^]^ It's interesting to note that CeO_2_ does not catalyze this reaction very well. MONMs including catalase,^[^ ^98^ ^]^ superoxidase,^[^ ^99^ ^]^ and phosphatase^[^ ^100^ ^]^ have also been shown, in addition to peroxidase and oxidase. More thorough reviews of nanozymes have been published by Wu^[^ ^102^ ^]^ and Huang.^[^ ^101^ ^]^ **Figure** **5** illustrates the steps involved in the synthesis of MONM using extracts from plants.

### 2.4 Quantum Dots Based Nanomaterials

**Figure 6:**
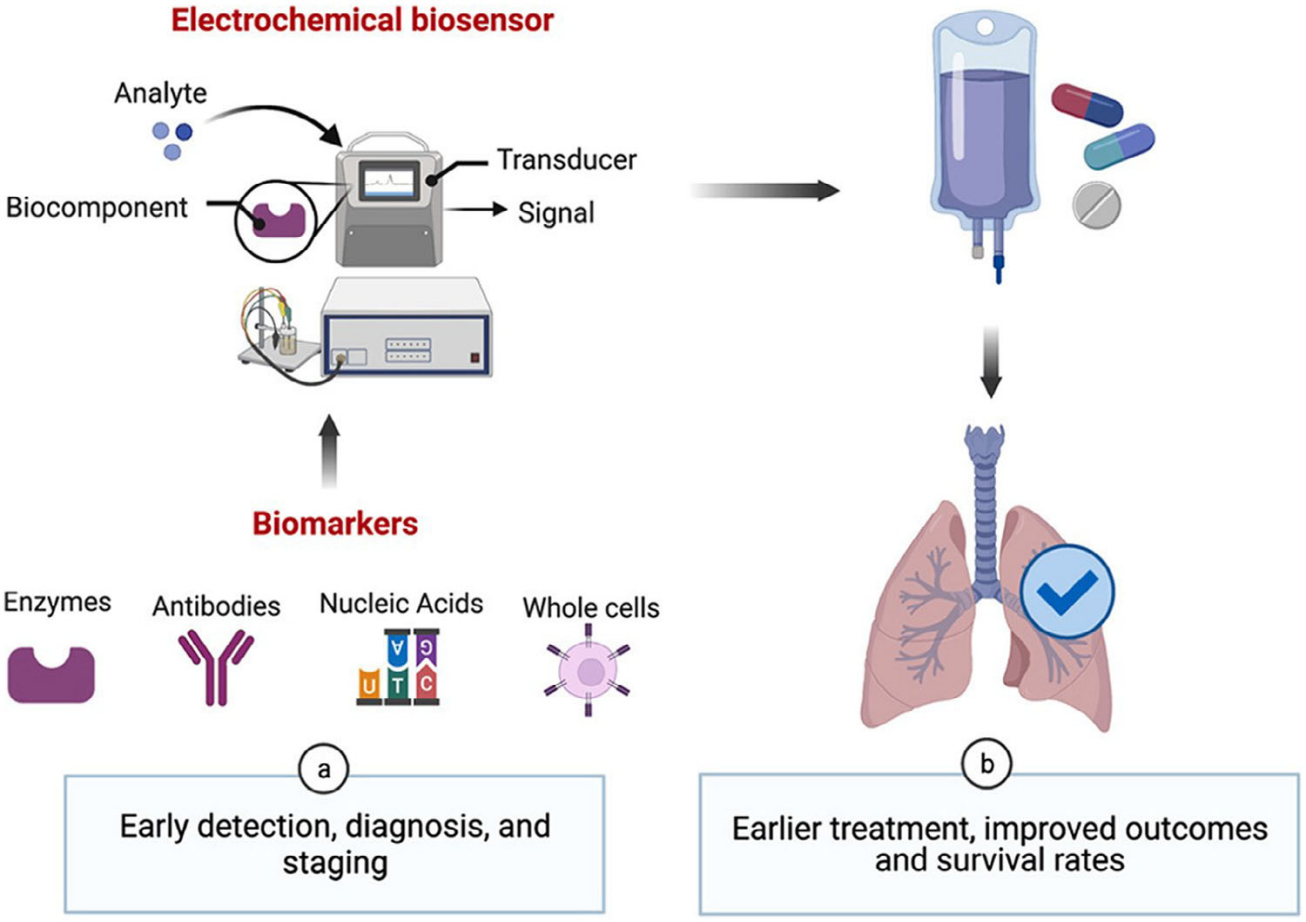
The a) early detection and b) earlier treatment. Reproduced with permission, Copyright 2021, Elsevier.^[^ ^107^ ^]^

Quantum dot‐based nanomaterials have emerged as a groundbreaking technology for sensor applications due to their unique optical and electronic properties. Quantum dots (QDs) are semiconductor nanocrystals that exhibit size‐dependent fluorescence, allowing precise control over their emission wavelengths by simply altering their size. This tunability, combined with their high photostability and broad absorption spectra, makes QDs highly effective for various sensing applications. In fluorescence‐based sensors, QDs can detect a wide range of analytes, including metal ions, biomolecules, and gases, by observing changes in their fluorescence intensity or wavelength. In electrochemical sensors, QDs enhance sensitivity and specificity by facilitating electron transfer processes, crucial for detecting substances like glucose and heavy metals. Optical sensors leverage the distinctive optical properties of QDs to identify environmental pollutants and toxins through changes in absorbance or reflectance.^[^ ^104^ ^]^ Additionally, QDs are used in photodetectors and biosensors, offering high sensitivity and fast response times for medical diagnostics and environmental monitoring. The continued advancement in the synthesis and functionalization of QDs promises to expand their applicability, making them a cornerstone of next‐generation sensor technology. Quantum yield (QY) is a crucial parameter for QDs, especially in sensing applications. A high QY means the QD is very bright and emits a lot of light for a given amount of excitation, while a low QY means it's less efficient and may lose energy through non‐radiative pathways (e.g., heat). In sensing, a high QY is generally desirable for sensitivity, detection limit and signal‐to‐noise ratio.^[^ ^105^ ^]^ Graphene quantum dots (GQDs) in conjunction with electrochemical biosensing systems have proven to be a suitable combination for cancer diagnostic approaches. This is especially true for identifying the alterations that promote the early stages of tumorigenesis and for detecting ultralow concentrations of biomarkers that differentiate between normal and malignant cells. GQDs, or graphene quantum dots, are a new type of zero‐dimensional semiconductor nanocrystals that vary in size from 1 to 50 nm and are organized in a honeycomb shape.^[^ ^106^ ^]^ These GQDs are similar in size to biomolecules, making them a perfect platform for studying biomolecules including proteins, cells, and viruses. GQDs work in perfect harmony with electrochemical sensors to provide a more targeted and sensitive platform for the identification of cancer biomarkers. Recent developments in the area of GQD‐based electrochemical sensors for early cancer detection were discussed by Tabish et al.^[^ ^107^ ^]^ The working of electrochemical sensor is depicted in **Figure** **6**.

**Figure 7:**
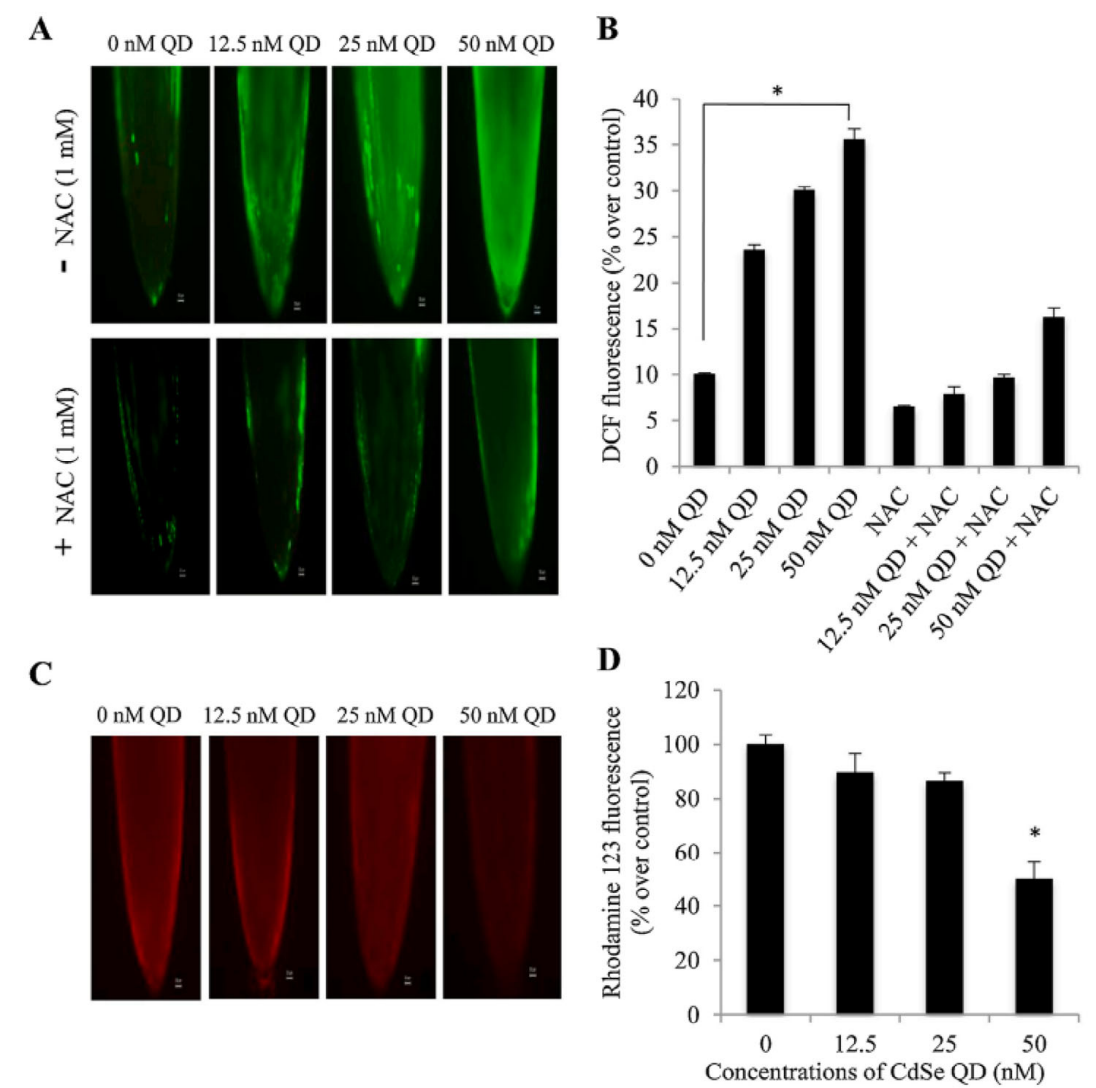
Allium cepa roots treated for three hours to varying concentrations of CdSe QDs and the resultant ROS production and impact on mitochondrial membrane potential. A) Locating ROS as indicated by a rise in DCF fluorescence; B) Bar graph demonstrating the measurement of increased DCF fluorescence in CdSe QD‐treated root tips and decreased DCF fluorescence in CdSe QD‐treated root tips due to NAC pretreatment. C) Rhodamine 123 fluorescence in CdSe QD‐treated root tips declines, indicating depolarisation of mitochondrial potential. D) Bar graph demonstrating Rhodamine 123 fluorescence quantification in root tips treated with CdSe QD. The data are shown as mean ± SEM (n = 3), with error bars that match SEM. One‐way ANOVA indicates that the data are significant in relation to the control at *p < 0.05. Bar scale: 50 µm. Nacetyl‐l‐cysteine, or NAC. Reproduced with permission, Copyright 2021, Elsevier.^[^ ^108^ ^]^

According to Banerjee et al.,^[^ ^108^ ^]^ QDs exhibited notable genotoxic effects linked to oxidative stress. This may be connected to the dose‐dependent increase in Cd retention in Allium roots, with the maximum absorption of CdSe QD occurring at 50 nm. The CdSe QD treatment‐induced oxidative stress triggered the activation of antioxidant enzymes (GPOD, GSH) and antioxidant scavengers (SOD, CAT). It was discovered that CdSe QD concentrations as low as 25 nm were cytotoxic and 50 nm CdSe QDs were genotoxic to the plant. Using the oxidation‐sensitive H2DCFDA fluorescent probe, the induction of ROS in *A. cepa* roots after a 3‐hour exposure to CdSe QDs was assessed; at all tested doses (12.5, 25, and 50 nm CdSe QD), a noticeable dose‐dependent increase in ROS production was seen. 50 nm CdSe QD had the highest fluorescence intensity (about 3.5 times higher than the control). N‐acetylcysteine (NAC) is a well‐known ROS inhibitor that was used to significantly reduce the formation of ROS in *A. cepa* roots generated by CdSe QD (**Figure** **7**A,B). This may support the production of ROS by CdSe QD in an indirect way. Fluorescent microscopy of *A. cepa* roots treated with CdSe QD and labeled with Rh123 demonstrated a significant collapse in the potential of the mitochondrial membrane caused by oxidative stress (Figure 7C,D). At lower doses (12.5 and 25 nm CdSe QD), there was no discernible difference in the fluorescence intensity compared to the control. Root fluorescence dramatically decreased at 50 nm CdSe QD (*p* < 0.05) with 90% quantum yield.

### 2.5 Other Nanomaterials

**Figure 8:**
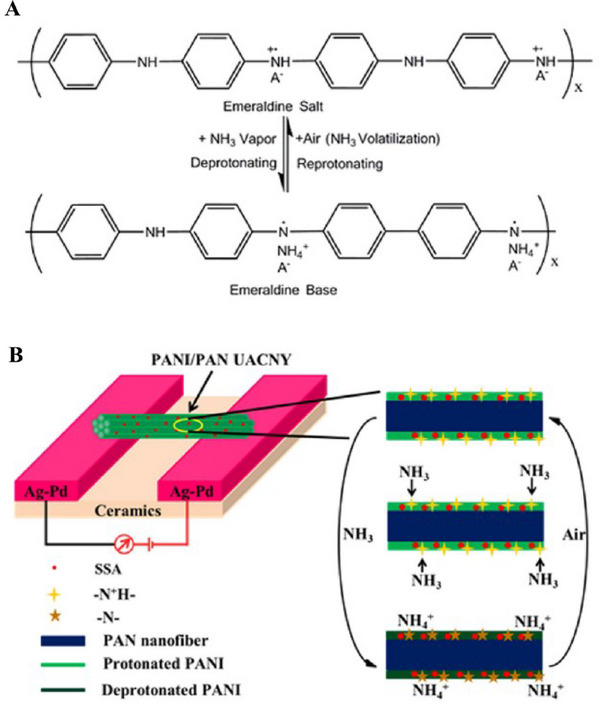
Mechanism of the PANI/PAN UACNY sensor's as‐prepared sense to NH_3_: A The PANI‐A− (emeraldine salt)‐PANI emeraldine base process of deprotonation and reprotonation. A− stands for the acid dopant SSA's anionic group; B) Diagrammatic representation of PANI/PAN UACNY sensor's sensitization process. Reproduced with permission, Copyright 2017, Elsevier.^[^ ^113^ ^]^

In the category of other materials, we have a lot of polymer‐based nanomaterials, composite‐based nanomaterials, metal–organic framework‐based nanomaterials, MXene‐based nanomaterials, etc. Polymer‐based nanomaterials have become a significant focus in the field of sensor applications due to their versatility, tunability, and unique properties.^[^ ^109^ ^]^ These nanomaterials, which can be engineered with precise control over their chemical and physical characteristics, offer a broad range of functionalities for detecting various analytes. Polymers can be synthesized to possess specific conductive, optical, and mechanical properties, making them suitable for diverse sensing mechanisms.^[^ ^110^ ^]^ In chemical sensors, polymer‐based nanomaterials can selectively interact with target molecules, producing detectable changes in electrical conductivity or fluorescence. This selectivity is often enhanced by incorporating functional groups or nanoparticles into the polymer matrix. In biosensors, biocompatible polymers can be designed to interact with biological molecules such as enzymes, antibodies, or DNA, enabling the detection of specific biomarkers for medical diagnostics.^[^ ^111^ ^]^ Additionally, polymer nanocomposites, which combine polymers with other nanomaterials like carbon nanotubes or metal nanoparticles, exhibit enhanced sensitivity and response times due to the synergistic effects of their components. The flexibility and processability of polymers also allow for the development of innovative sensor designs, including flexible and wearable sensors for real‐time monitoring of physiological parameters.^[^ ^112^ ^]^ Moreover, the environmental stability and durability of polymer‐based nanomaterials make them ideal for long‐term applications in environmental monitoring and industrial processes. As research progresses, the continued refinement and functionalization of polymer nanomaterials promise to advance their role in creating highly sensitive, selective, and durable sensors for a wide array of applications. A number of factors should be taken into account in order to understand why PANI/PAN UACNY's NH_3_ sensing performance has improved. In order to better illustrate the sensing process, **Figure** **8** presents a simplified model.^[^ ^113^ ^]^ As shown in Figure 8A, the deprotonating‐reprotonating process of PANI‐A (emeraldine salt)‐PANI emeraldine base may be used to understand the mechanism of this gas sensor, which is derived from the diffusion of NH_3_ into the PANI/PAN UACNY and the interaction of NH_3_ with PANI. When ammonia molecules come into contact with NH_3_, their lone pair electrons allow them to form a coordination bond with the dopant proton's free atomic orbital. As a result of this process, the PANI nitrogen atoms deprotonate, causing the polaron, one of the charge carriers, to vanish and increasing electrical resistance. In the opposite cycle, NH_3_ is reversibly transformed into PANI salt by desorbing and diffusing back from the PANI base. The electrical conductance increases when the concentration of NH_3_ surrounding the nanostructured PANI base decreases because more NH_3_ molecules are released, freeing up more current carriers in the process. In addition to the above‐mentioned conducting polymer PANI‐A‐ (emeraldine salt) sensing mechanism, the structure of the PANI/PAN UACNY sensor in Figure 8B provides an additional explanation for the good sensing property of PANI/PAN UACNY to NH_3_ with a quick response and high sensitivity.

Nanomaterials offer exciting possibilities for sensing applications, their practical implementation faces several limitations and challenges such as stability and reproducibility, selectivity and interference, sensitivity and detection limit, cost and scalability, etc.^[^ ^114^ ^]^ Stability and reproducibility are crucial for reliable sensor performance. In nanoparticle‐based sensors, stability can be affected by factors like aggregation, oxidation, and environmental degradation. Reproducibility can be influenced by variations in nanoparticle synthesis, sensor fabrication, and measurement conditions.^[^ ^115^ ^]^ Addressing these challenges requires careful control over nanomaterial properties, sensor design, and operating conditions. In nanoparticle‐based sensors, selectivity can be enhanced by surface modification with specific ligands or receptors that interact selectively with the target analyte. Interference can be minimized by optimizing the sensor design, controlling the measurement conditions, and employing appropriate data analysis techniques.^[^ ^116^ ^]^ Sensitivity and detection limit are two key parameters that determine the performance of a sensor. Sensitivity refers to the smallest change in the analyte concentration that the sensor can detect, while the detection limit is the lowest concentration of the analyte that can be reliably measured by the sensor.^[^ ^117^ ^]^ Nanoparticle‐based sensors can exhibit high sensitivity due to the unique properties of nanomaterials, such as their high surface area and ability to interact strongly with target analytes. However, achieving low detection limits can be challenging due to factors like background noise and interference from other substances in the sample.^[^ ^118^ ^]^ The synthesis and processing of high‐quality nanomaterials can be expensive, limiting their widespread adoption. Scaling up the production of nanomaterial‐based sensors while maintaining their performance and quality can be a challenge.^[^ ^119^ ^]^

## 3 Performance and Validation

**Figure 9:**
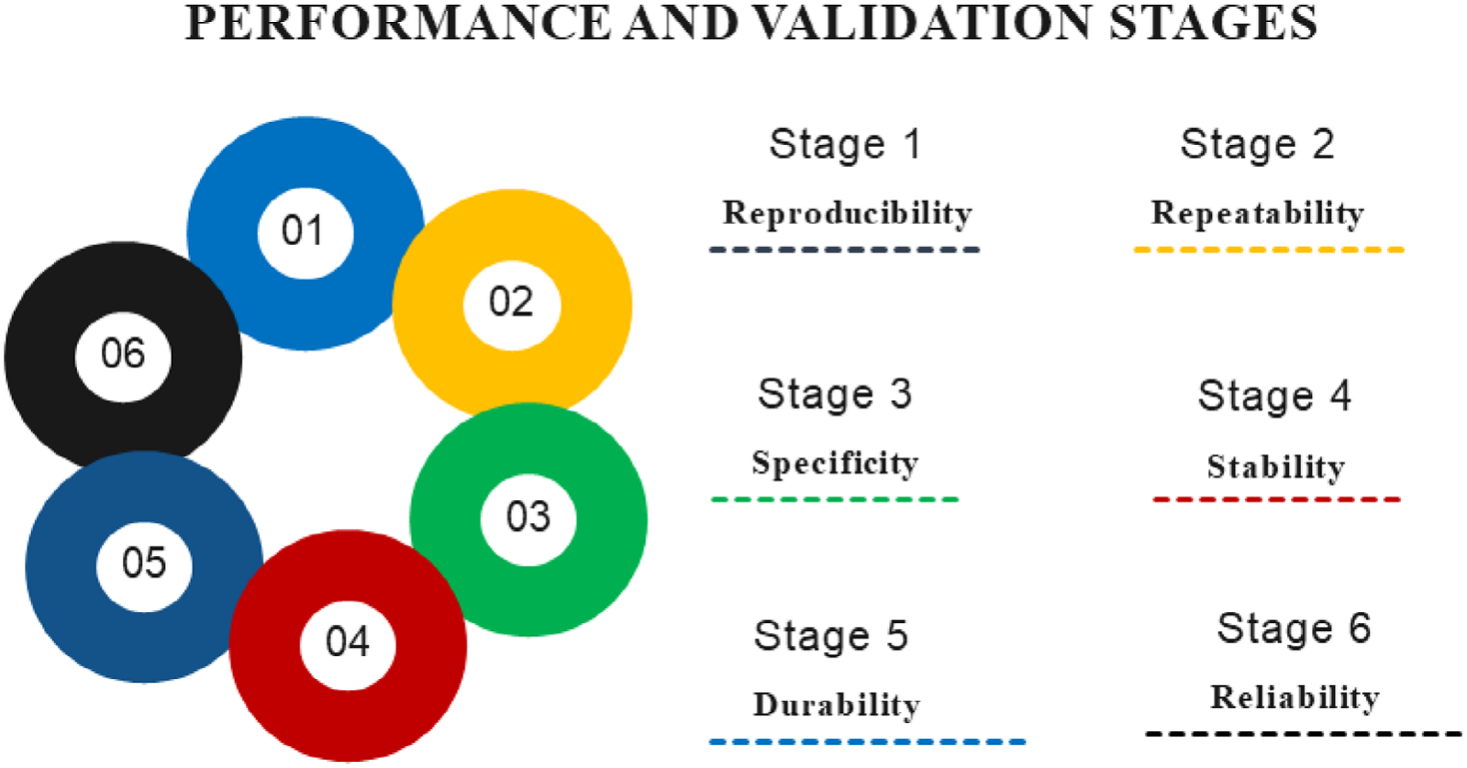
Steps involved in the validation of results.

Performance and validation (**Figure** **9**) of a sensor are essential processes to ensure its accuracy, reliability, and suitability for its intended application. Performance evaluation involves testing the sensor's ability to accurately measure and respond to the target variables under various conditions. This includes assessing its sensitivity, specificity, and response time to ensure it meets the required performance standards. **Table** **1** demonstrates the comparison analysis of the already reported work. Validation, on the other hand, involves comparing the sensor's readings against established benchmarks or reference standards to confirm its accuracy and consistency. This process may include laboratory testing, field trials, and real‐world application scenarios to verify that the sensor performs reliably across different environments and conditions.^[^ ^132^ ^]^ Effective performance and validation are crucial for establishing trust in the sensor's data and ensuring it meets regulatory and industry standards, ultimately impacting its commercial success and user acceptance.

**Table 1:** Comparison analysis of the previous reported work.

Target	Nanomaterial	Detection limit	System	Sensing probe	Reference
H_2_O_2_	AgNCs	2 µm	AgNCs/PB/PET	Glucose oxidase	[120]
Glucose	Au/MoS_2_/Au nanofilm	10 nm	GO_x_/Au/MoS_2_ /Au/PI	Glucose oxidase	[121]
DA	GO/UCNPs	≈1 pm	GO/Aptamer/SW‐UCNPs	DA‐specific aptamer	[122]
PSA, EphA2	UCNPs	89 pg mL^−1^ (PSA), 400 pg mL^−1^ (EphA2)	Highly doped UCNPs	PSA antibody, EphA2 antibody	[123]
Carcinoembrynic antigen (CEA)	Ti_3_C_2_	0.018 pg mL^−1^	Anti‐CEA/f‐Ti_3_C_2_ /GC	Carcino embryonic antibody monoclonal	[124]
VP40	MoS_2_	2 fM	VP40/BSA/MoS_2_ /Au electrode	VP40 antibody	[125]
Nucleic acid	MWCNT	1 fM	pDNA/MWCNT/ITO	Amine terminated DNA	[126]
DA, Hg^2+^, Cu^2+^, Cd^2+^	GQDs	140 nm (Cd^2+^), 120 nm (DA), 32 nm (Cu^2+^), 0.3 µM (Hg^2+^)	GQDs/VMSF	OH‐GQDs, NH_2_ ‐GQDs	[127]
DNA	CDs	0.16 nm	DNA/Probe/CDs/AuSPE	Unmodified oligonucleotides	[128]
Eosinophil cationic protein (ECP)	Au@Fe_3_O_4_	0.3 nm	Hep‐Au@Fe_3_O_4_	Hep‐Au@Fe_3_O_4_	[129]
HIV p24	PtNPs	0.8 pg mL^−1^	Ab‐PtNPs/HIV p24/Nanobody‐biotin/ polystrept abidin	HIV p24 antibody/ PtNPs conjugates	[130]
c‐PSA	AuNPs/magnetic NPs	0.15 ng mL^−1^ (c‐PSA)	PSA/MGITC@AuNPs/ magnetic NPs	c‐PSA antibody	[131]
f‐PSA	AuNPs/magnetic NPs	0.012 ng mL^−1^ (f‐PSA),	PSA/MGITC@AuNPs/ magnetic NPs	f‐PSA antibody	[131]

### 3.1 Reproducibility

**Figure 10:**
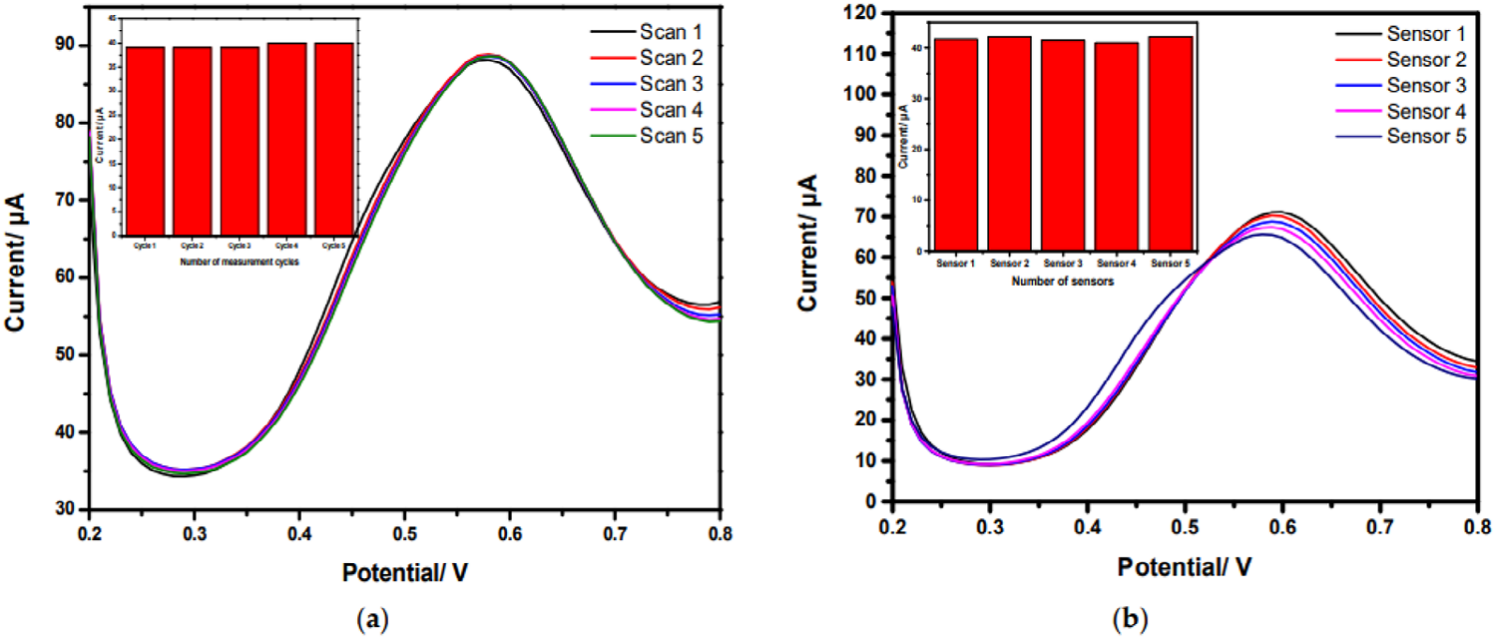
The electrode's reproducibility was examined in 0.001 m of nitrite and 0.1 m of PBS (pH 6.5) a) inter‐study and b) intra‐study. Reproduced under Creative Common CC BY license, 2022 @ MDPI.^[^ ^136^ ^]^

Reproducibility is a critical factor in the development and commercialization of sensor technologies, particularly when transitioning from lab to market. It refers to the sensor's ability to consistently produce the same results under identical conditions across different instances and batches. Achieving high reproducibility ensures that the sensor can reliably perform its intended function, whether in industrial automation, healthcare diagnostics, or environmental monitoring, without significant variation in output.^[^ ^133^ ^]^ This consistency is crucial for building trust among end‐users and regulatory bodies, as well as for maintaining quality control during mass production. To achieve reproducibility, meticulous attention must be paid to the materials used, the manufacturing processes, and the calibration methods employed. Each step in the sensor's production must be standardized and thoroughly tested to ensure that every unit functions as expected, regardless of when or where it is produced. Additionally, rigorous testing under various environmental and operational conditions should be conducted to confirm that the sensor's performance remains stable across different applications and over time. Reproducibility not only underpins the technical success of the sensor but also plays a key role in its commercial viability, as inconsistent performance can lead to product recalls, increased costs, and damage to the brand's reputation.^[^ ^134^, ^135^ ^]^ Therefore, ensuring reproducibility is a foundational aspect of the development process that directly impacts the sensor's market acceptance and long‐term success. According to Talbi et al.,^[^ ^136^ ^]^ a highly sensitive electrochemical sensor based on carbon screen‐printed electrodes (CSPE) with gold nanoparticles (AuNPs, ≈12 nm) surface‐modified by photochemical synthesis is suggested for nitrite detection. DPV curves were initially recorded using a single CSPE/AuNPs‐PEI electrode in five consecutive measurements at a nitrite concentration of 0.001 m with 0.1 m of PBS solution at pH 6.5 in order to assess the repeatability of the sensor's response. **Figure** **10**a illustrates that following five successive observations, the DPV curves' form and corresponding current signals stay constant. An intra‐study based on evaluating five distinct electrodes made under the same experimental settings, as shown in Figure 10b, demonstrates an RSD less than 10%. As a result, the recommended sensor for nitrite measurement has high repeatability.

### 3.2 Repeatability

Repeatability is a fundamental aspect of sensor performance, particularly in applications where precision and reliability are paramount. It refers to the sensor's ability to produce the same measurement results consistently when subjected to the same conditions, including identical inputs, environment, and operational settings, over multiple trials. High repeatability ensures that the sensor can be trusted to provide accurate and reliable data, which is crucial in fields like medical diagnostics, environmental monitoring, and industrial automation, where decisions based on sensor data can have significant consequences. To achieve excellent repeatability, the sensor design must minimize variability due to factors such as noise, drift, and environmental interference. This involves selecting high‐quality, stable materials and components, as well as implementing robust calibration procedures. The manufacturing process must also be tightly controlled to ensure that each sensor is produced to the same specifications, thereby reducing unit‐to‐unit variability. Furthermore, repeatability testing under different scenarios is essential to confirm that the sensor performs consistently across a range of conditions, including varying temperatures, pressures, and humidity levels. Repeatability is not only a technical requirement but also a key selling point, as customers rely on sensors that can consistently deliver the same results, ensuring the accuracy and dependability of their operations or products. A sensor that lacks repeatability may lead to erroneous data, which can result in costly errors, inefficiencies, or even safety hazards.^[^ ^137^ ^]^ Therefore, emphasizing and ensuring repeatability is critical to the sensor's success, as it directly impacts the credibility, usability, and commercial potential of the technology.

### 3.3 Specificity

**Figure 11:**
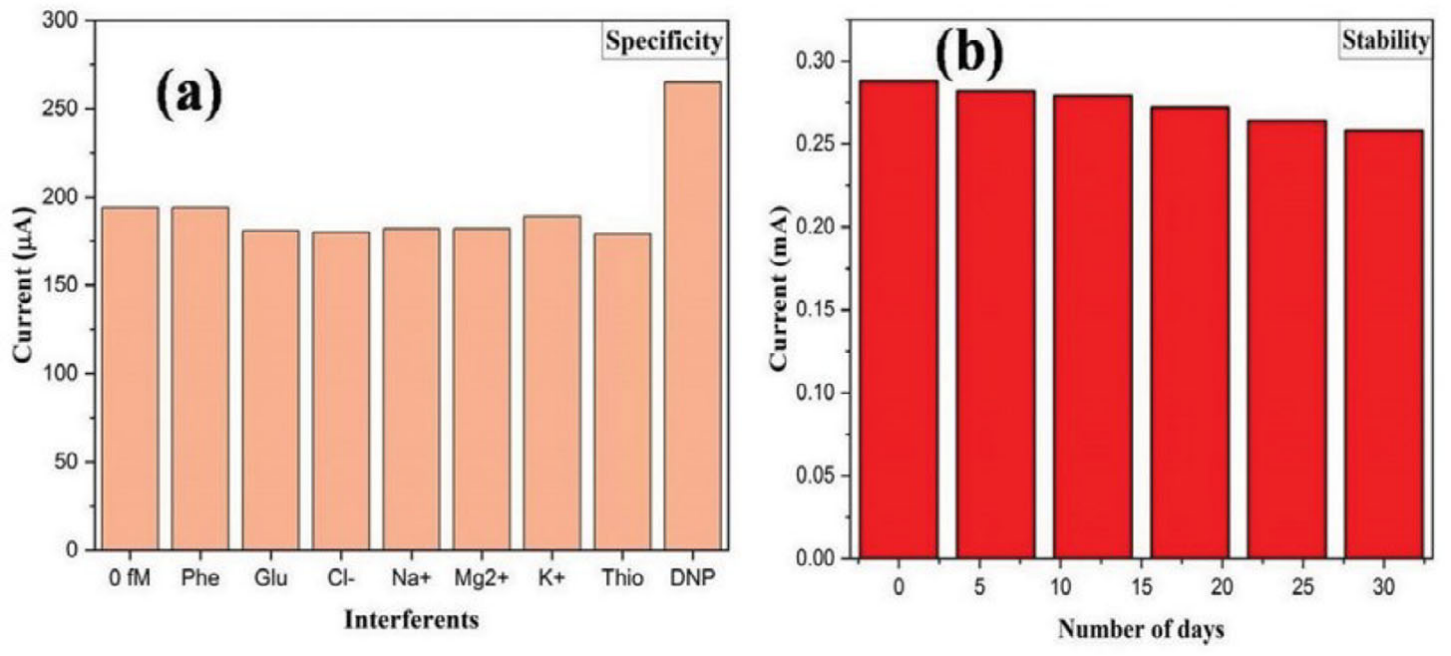
a) Specificity of Bi_2_O_3_ electrode and b) Stability of Bi2O3 electrode. Reproduced with permission, Copyright 2022, Elsevier.^[^ ^139^ ^]^

Specificity is a crucial attribute of sensor performance, particularly in applications where distinguishing between closely related signals or substances is essential. It refers to the sensor's ability to accurately identify and measure a specific target analyte or signal in the presence of other potentially interfering substances. High specificity ensures that the sensor can selectively detect the intended target without being influenced by background noise or other similar entities, which is vital in fields such as medical diagnostics, environmental monitoring, and chemical detection. For instance, in a medical context, a sensor with high specificity can distinguish between different biomarkers, leading to accurate diagnoses and treatment plans. Achieving high specificity requires careful design of the sensor's recognition elements, such as using selective binding sites or highly specific chemical reactions that target the desired analyte. Additionally, advanced signal processing techniques may be employed to further enhance the sensor's ability to discriminate between the target and non‐target substances. Rigorous testing in real‐world conditions is necessary to validate the sensor's specificity, ensuring it performs reliably in complex environments where various interfering factors may be present. Specificity not only enhances the accuracy of the sensor but also reduces the likelihood of false positives or negatives, which can have significant implications in critical applications.^[^ ^138^ ^]^ A sensor lacking specificity may lead to incorrect readings, resulting in misguided decisions, wasted resources, or even health and safety risks. Therefore, specificity is a key factor in the sensor's design and performance, directly influencing its effectiveness, reliability, and marketability. Ensuring that a sensor has high specificity is essential for its acceptance and success in its intended application. In order to electrochemically detect 2,4‐Dinitrophenol (2,4‐DNP), Balwan et al.^[^ ^139^ ^]^ used one‐dimensional Bismuth (III) oxide (Bi_2_O_3_) nanostructures as selective materials. The specificity of the Bi_2_O_3_ electrode is shown in **Figure** **11**a and stability is shown in Figure 11b.

### 3.4 Stability

Stability is a vital characteristic of sensor performance, reflecting its ability to maintain consistent functionality and accuracy over time under various environmental and operational conditions. A stable sensor ensures reliable data output, even when subjected to changes in temperature, humidity, mechanical stress, or prolonged use, making it indispensable in applications like industrial automation, environmental monitoring, and healthcare. High stability is crucial because it directly impacts the sensor's long‐term reliability and reduces the need for frequent recalibration or maintenance, which can be costly and time‐consuming. Achieving stability requires a careful selection of durable materials and components that are resistant to degradation and wear. The sensor's design must also account for potential environmental influences, incorporating protective measures such as shielding, insulation, or temperature compensation to mitigate these effects.^[^ ^140^ ^]^

Nanoparticle‐based sensors hold immense potential for various applications due to their high sensitivity and unique properties. However, ensuring their stability and reproducibility is crucial for reliable and consistent performance. Various methods are employed to enhance the stability and reproducibility of sensors developed using nanoparticles such as surface modification and functionalization, matrix stabilization, device integration and packaging, and advanced synthesis techniques.^[^ ^141^ ^]^ Applying capping agents or ligands to the nanoparticle surface can prevent aggregation, oxidation, and chemical degradation. These agents provide a protective layer, enhancing stability. Introducing specific functional groups onto the nanoparticle surface enables selective interaction with target analytes. This enhances sensitivity and reduces interference, improving reproducibility.^[^ ^142^ ^]^ Incorporating nanoparticles into a stable matrix, such as a polymer or sol‐gel, can provide physical support and prevent aggregation. The matrix also protects nanoparticles from harsh environments, improving stability. This technique allows for the controlled deposition of nanoparticles and other materials, creating a structured architecture that enhances stability and reproducibility.^[^ ^143^ ^]^ Integrating nanoparticles into a well‐designed device protects them from external factors and ensures proper contact with the target analyte. Packaging plays a vital role in preserving sensor stability during storage and use. It should protect the sensor from environmental influences and mechanical stress. Atomic Layer Deposition enables precise coating of nanoparticles with thin films, enhancing stability and providing additional functionality.^[^ ^144^ ^]^ Integrating nanoparticle‐based sensors with microfluidic systems allows for precise control of sample delivery and reaction conditions, improving reproducibility. By employing these methods, the stability and reproducibility of nanoparticle‐based sensors can be significantly enhanced paving the way for their widespread use in various fields.^[^ ^145^ ^]^

### 3.5 Durability

Durability is a critical attribute of sensor performance, particularly for applications in harsh or demanding environments where the sensor must withstand physical stress, extreme temperatures, moisture, and chemical exposure over extended periods. A durable sensor maintains its functionality and accuracy despite these challenging conditions, making it essential for industries such as aerospace, automotive, industrial automation, and environmental monitoring.^[^ ^146^ ^]^ Durability ensures that the sensor can operate reliably with minimal degradation or failure, which is vital for reducing downtime, maintenance costs, and the need for frequent replacements. Achieving high durability requires the use of robust materials that can resist wear and tear, corrosion, and mechanical impact. The sensor's design must also account for factors such as vibration, shock, and thermal cycling, incorporating features like rugged casings, flexible circuitry, or protective coatings to shield sensitive components. Durability testing is an integral part of the development process, where sensors are subjected to accelerated aging tests, mechanical stress tests, and environmental simulations to assess their resilience. A sensor that lacks durability may fail prematurely, leading to inaccurate data, operational disruptions, and potentially significant financial losses or safety hazards. In contrast, a durable sensor provides long‐term reliability, even in the most demanding conditions, which enhances its value proposition and makes it a preferred choice for critical applications. Durability is not only a measure of the sensor's physical robustness but also its ability to maintain performance over time, ensuring consistent output without significant deterioration.^[^ ^147^ ^]^ Thus, designing for durability is essential for creating sensors that meet the rigorous demands of industrial and commercial use, ensuring they deliver dependable performance throughout their lifecycle and thereby earning the trust of users in high‐stakes environments.

### 3.6 Reliability

Reliability is a cornerstone of sensor performance, defining its ability to consistently deliver accurate and dependable results over its operational lifetime. In critical applications such as healthcare diagnostics, industrial automation, aerospace, and environmental monitoring, sensor reliability is paramount because it directly impacts the quality of data and, consequently, the decisions and actions taken based on that data. A reliable sensor must perform its intended function with minimal errors, downtime, or maintenance, even when exposed to varying conditions such as temperature fluctuations, humidity, mechanical vibrations, and electrical noise. Achieving high reliability involves a combination of rigorous design, quality manufacturing processes, and thorough testing.^[^ ^148^ ^]^ The sensor's components and materials must be carefully selected to ensure they can endure operational stresses without degrading. Additionally, redundancy in critical parts, error‐checking algorithms, and fault‐tolerant designs may be incorporated to enhance reliability. Comprehensive testing under real‐world conditions is essential to validate the sensor's reliability, including long‐term testing to simulate years of use, and stress testing to push the sensor to its operational limits. Reliability also encompasses the sensor's ability to maintain calibration and accuracy over time, requiring minimal recalibration or adjustment. A sensor that lacks reliability can lead to frequent failures, inaccurate readings, and ultimately a loss of trust from users, which can be costly and dangerous in sensitive applications. In contrast, a highly reliable sensor enhances user confidence, reduces the need for constant monitoring or replacement, and ensures that systems relying on the sensor can function smoothly without interruption. Ultimately, reliability is a key factor that determines the overall effectiveness and success of a sensor in any application, making it an essential focus during the design, development, and production processes.^[^ ^149^ ^]^

Nanomaterials possess unique properties that make them ideal for enhancing the robustness, versatility, and sensitivity of sensors. Nanomaterials possess a large surface area compared to their volume. This provides more active sites for interaction with target analytes, leading to enhanced sensitivity. Even small amounts of analyte can interact with a significant portion of the sensor material, producing a detectable signal.^[^ ^150^ ^]^ At the nanoscale, the electronic and optical properties of materials can differ significantly from their bulk counterparts due to quantum confinement effects. These effects can be tuned by controlling the size and shape of nanomaterials, allowing for the development of sensors with specific functionalities and sensitivities. The high surface area and unique electronic properties of nanomaterials can lead to enhanced chemical reactivity. This can improve the interaction between the sensor and the target analyte, leading to stronger signals and faster response times. The properties of nanomaterials can be tailored by controlling their size, shape, composition, and surface functionality. This tunability allows for the development of sensors that are highly selective for specific analytes and can operate in a variety of environments. Nanomaterials can be used in a variety of sensing mechanisms, including electrochemical, optical, and mechanical sensing. This versatility allows for the development of sensors for a wide range of applications, from detecting chemical pollutants to monitoring biological processes. Nanomaterials can be easily integrated into micro‐ and nano‐scale devices, enabling the development of miniaturized sensors with high sensitivity and low power consumption. This is particularly important for applications in portable devices and implantable sensors.^[^ ^151^ ^]^

## 4 Market Research

Market research and feasibility analysis of a sensor are critical steps in determining its potential success in the market. Market research involves gathering and analyzing data on industry trends, customer needs, and competitive products to identify the demand for the sensor and its target market. This process helps in understanding the size and growth potential of the market, customer preferences, and the competitive landscape. Feasibility analysis, on the other hand, assesses the practical aspects of developing and commercializing the sensor. It examines technical feasibility, ensuring the sensor can meet performance requirements and be produced at scale. Operational feasibility looks at integration with existing systems and maintenance needs, while financial feasibility evaluates the costs versus potential revenue, ensuring a profitable return on investment. Regulatory and legal feasibility is also considered, ensuring compliance with industry standards and protecting intellectual property. Together, market research and feasibility analysis provide a comprehensive understanding of the sensor's viability, guiding informed decision‐making and strategic planning.

### 4.1 Identify Potential Markets

**Figure 12:**
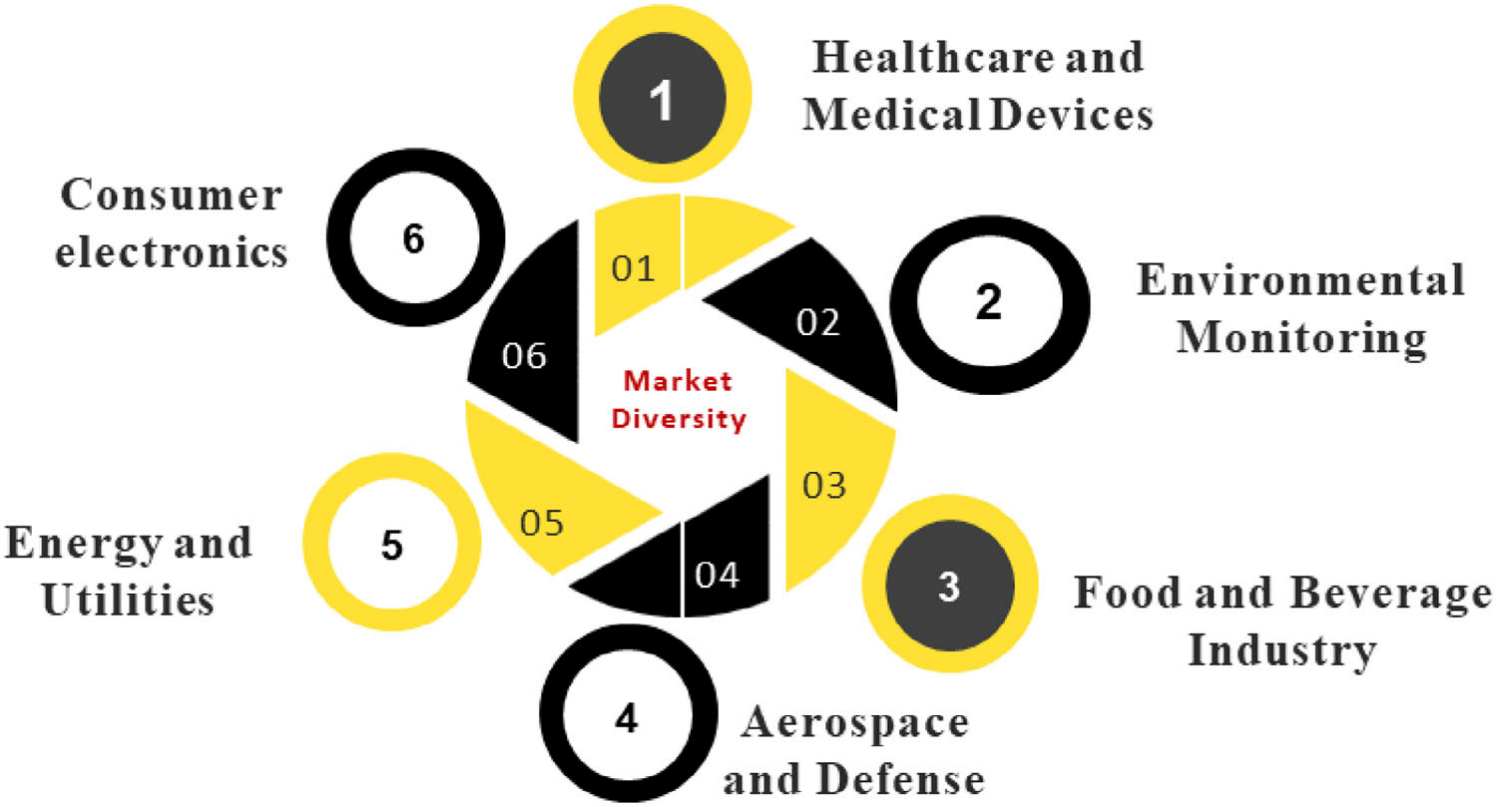
Schematic view of potential markets of sensors.

Identifying potential markets for sensors requires considering the specific capabilities of the sensor technology and the industries that would benefit most from those capabilities. In this section, several key markets where sensors are in high demand are described (**Figure** **12**).

#### 4.1.1 Healthcare and Medical Devices

**Figure 13:**
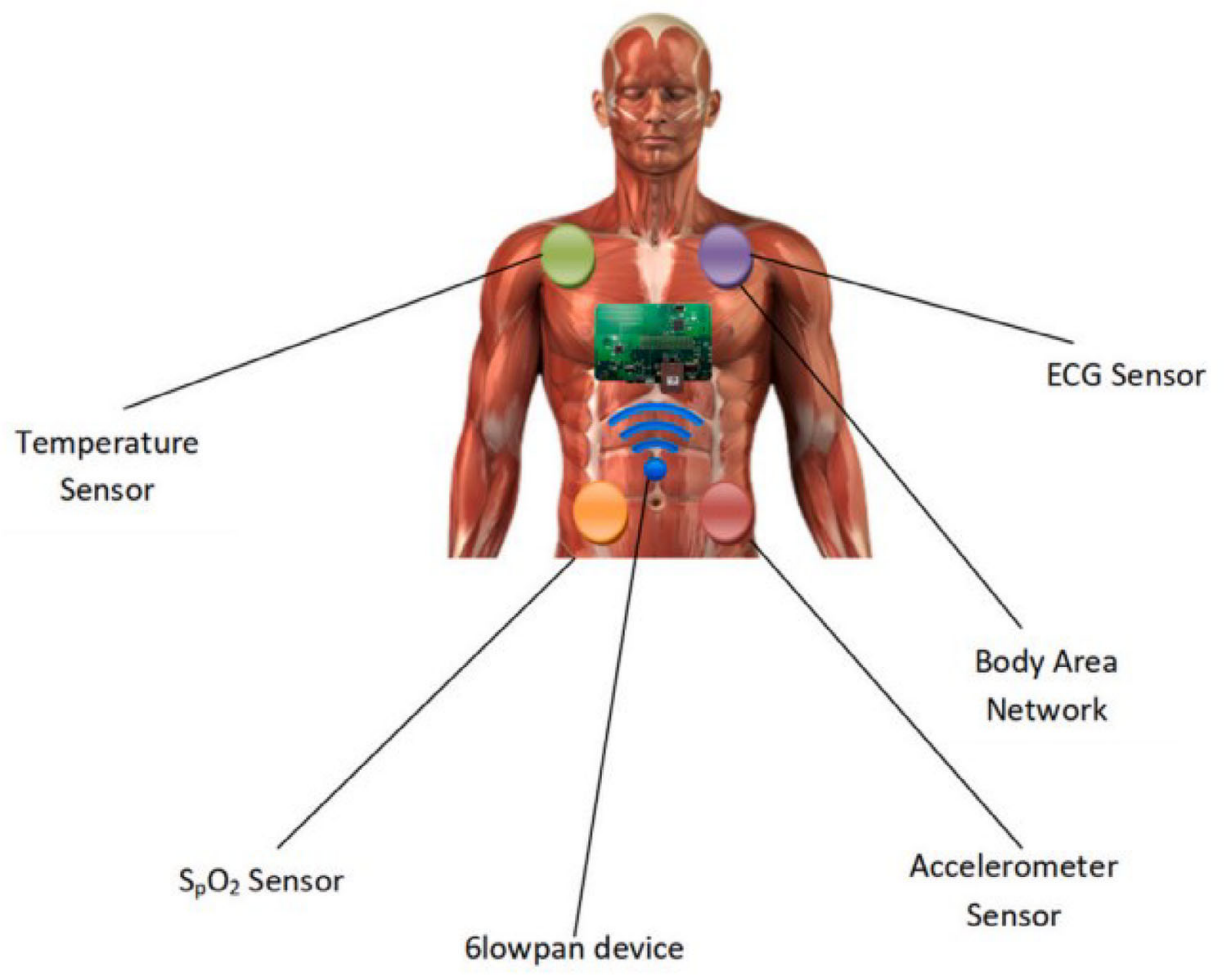
Various sensors attached with human patient body. Reproduced with permission, Copyright 2023, Elsevier.^[^ ^153^ ^]^

The healthcare and medical devices market represents a dynamic and rapidly expanding sector for sensor technologies, driven by the growing demand for advanced diagnostics, personalized medicine, and improved patient outcomes. Sensors play a crucial role in this market, enabling innovations across various applications, from wearable health monitors and in‐home medical devices to sophisticated diagnostic tools and surgical equipment. The increasing prevalence of chronic diseases, an aging population, and a global shift toward preventive healthcare have amplified the need for sensors that can monitor vital signs, detect early disease markers, and provide real‐time health data. The patient has a sensor device affixed to their body to continuously monitor and gather medical data. Such parameters as insulin, peripheral and internal heart temperatures, pulse rate, SpO_2_, and many more are measured by these sensors. **Figure** **13** indicates that IEEE 802.15.4 has classified two types of devices as Sensor Networks: Full Function Devices and Lower devices. Peer‐to‐peer topology with multi‐hop connections is supported by FFDs, which provide all system domains.^[^ ^152^ ^]^ RFD devices can only monitor physicochemical properties and carry out basic tasks in the network of stars. Every PAN coordinator is normally in charge of controlling the PAN and is also in charge of configuring and maintaining it.^[^ ^153^ ^]^ Fitness trackers, smartwatches, and continuous glucose monitors are examples of wearable technology that is gaining popularity and enabling people to take charge of their health. In clinical settings, sensors are integral to patient monitoring systems, ensuring continuous tracking of parameters like heart rate, blood pressure, oxygen saturation, and temperature, which are critical for managing acute and chronic conditions. Moreover, in vitro diagnostic devices rely heavily on biosensors for rapid, accurate detection of biomarkers, pathogens, and other analytes, facilitating early diagnosis and treatment. The rise of telemedicine and remote patient monitoring, accelerated by the COVID‐19 pandemic, has further highlighted the importance of reliable and precise sensors in delivering healthcare services beyond traditional clinical environments. This market is also witnessing significant innovation in implantable sensors that provide continuous, long‐term monitoring within the body, offering new possibilities for managing diseases like diabetes, cardiovascular conditions, and neurological disorders. As the healthcare industry increasingly integrates digital technologies, sensors are central to the development of smart medical devices that can improve patient outcomes, reduce healthcare costs, and enhance the overall efficiency of healthcare systems. Regulatory considerations, including FDA approval and compliance with international standards, are critical in this market, as they ensure the safety and effectiveness of sensor‐enabled medical devices. With continuous advancements in sensor technology, such as miniaturization, improved sensitivity, and wireless connectivity, the healthcare and medical devices market is poised for sustained growth, offering immense opportunities for innovation and commercialization.

#### 4.1.2 Environmental Monitoring

**Figure 14:**
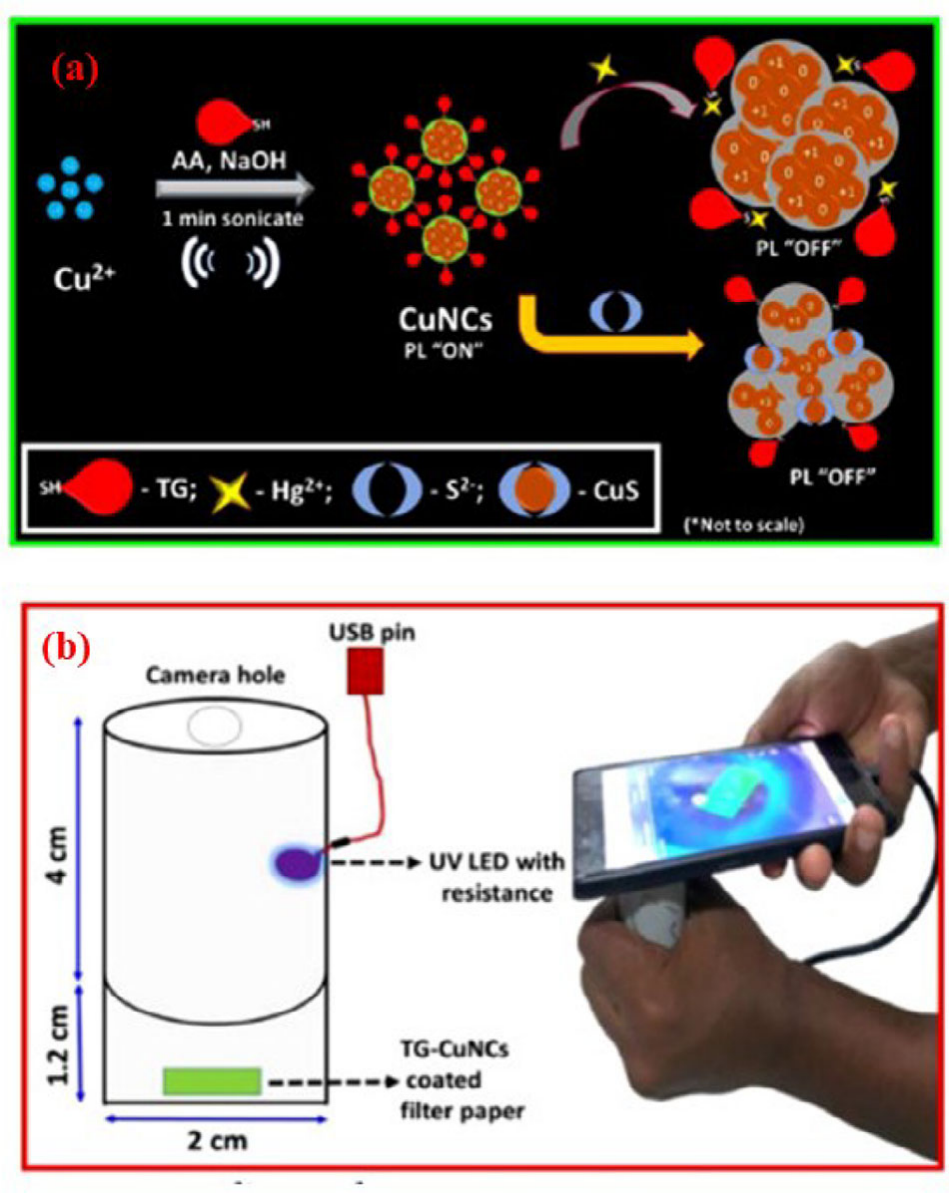
A useful method for detecting S^2−^ ions and Hg^2+^. a) mechanism of detection based on rapid production Cu NCs. b) Connecting the smartphone to the detecting system. Reproduced with permission, Copyright 2020, Elsevier.^[^ ^161^ ^]^

The environmental monitoring market is a critical and rapidly growing sector for sensor technologies, driven by the increasing global focus on sustainability, regulatory compliance, and the need to address environmental challenges such as climate change, pollution, and resource management. Sensors in this market are essential tools for detecting and quantifying various environmental parameters, including air and water quality, soil conditions, and atmospheric changes.^[^ ^154^, ^155^, ^156^ ^]^ Governments, industries, and environmental organizations rely on these sensors to monitor pollutants, track ecosystem health, and ensure compliance with environmental regulations. Air quality sensors, for instance, are increasingly deployed in urban areas to measure levels of pollutants like particulate matter, carbon dioxide, and volatile organic compounds, providing real‐time data that helps mitigate public health risks and inform policy decisions. In water quality monitoring, sensors are used to detect contaminants such as heavy metals, pesticides, and pathogens, ensuring the safety of drinking water supplies and the health of aquatic ecosystems. The rise of smart cities has further propelled the demand for environmental sensors, as urban planners integrate them into infrastructure to monitor environmental conditions and improve the quality of life for residents. Additionally, the agriculture sector utilizes soil and weather sensors to optimize crop production and water usage, contributing to more sustainable farming practices. The increasing severity of natural disasters, such as wildfires, floods, and hurricanes, has also highlighted the need for robust environmental monitoring systems that can provide early warnings and support disaster response efforts. Technological advancements, including the development of low‐cost, low‐power, and wireless sensors, have expanded the deployment of monitoring systems in remote and challenging environments, enabling more comprehensive and continuous data collection.^[^ ^157^, ^158^, ^159^ ^]^ Moreover, the integration of sensor data with IoT platforms and cloud computing has enhanced the ability to analyze and share environmental data, leading to more informed decision‐making and proactive environmental management. As global awareness of environmental issues continues to grow, and as regulatory frameworks become more stringent, the demand for innovative and reliable environmental sensors is expected to increase, making this market a vital area for ongoing research, development, and investment. Over the past five years, Zhu et al.^[^ ^160^ ^]^ have compiled the latest developments in nanomaterial‐based chemiluminescence in environmental pollutant analysis for common dangerous compounds, such as pesticides, antibiotics, and inorganic ions, among others. Finally, to encourage the development of environmental analysis, the main obstacles and future opportunities in this fascinating field are also addressed. More recently, the optimization of the Cu nanocluster manufacturing method was the main emphasis of Maruthupandi et al.^[^ ^161^ ^]^ The primary novelty reported in the paper is fast synthesis (1 min) using sonication. Copper nitrate, ascorbic acid, and 1‐Thio‐β‐D‐glucose in basic medium (NaOH) were utilized for the synthesis. With detection limits of 1.7 nm and 1.02 nm for mercury and Sulphur, respectively, these highly fluorescent nanoclusters can detect Hg^2+^ and S^2−^ ions by a fluorescence quenching phenomenon that is directly proportional to the analyte concentration. Environmental pollution is mostly caused by the S^2−^ anion, which is also linked to Alzheimer's disease and cirrhosis.^[^ ^162^ ^]^ The ease with which the task may be integrated with a smartphone is another innovation. **Figure** **14** illustrates the mechanism the scientists created in which the nanoclusters are deposited on a reactive strip (filter paper) and subsequently brought into contact with the problematic material. A UV LED built within a PVC tube and linked to a smartphone is used to take the reading. Recoveries for Hg^2+^ and S^2−^ in actual well and river water samples ranged from 98.5% to 101.4% and 99% to 101%, respectively.

#### 4.1.3 Food and Beverage Industry

**Figure 15:**
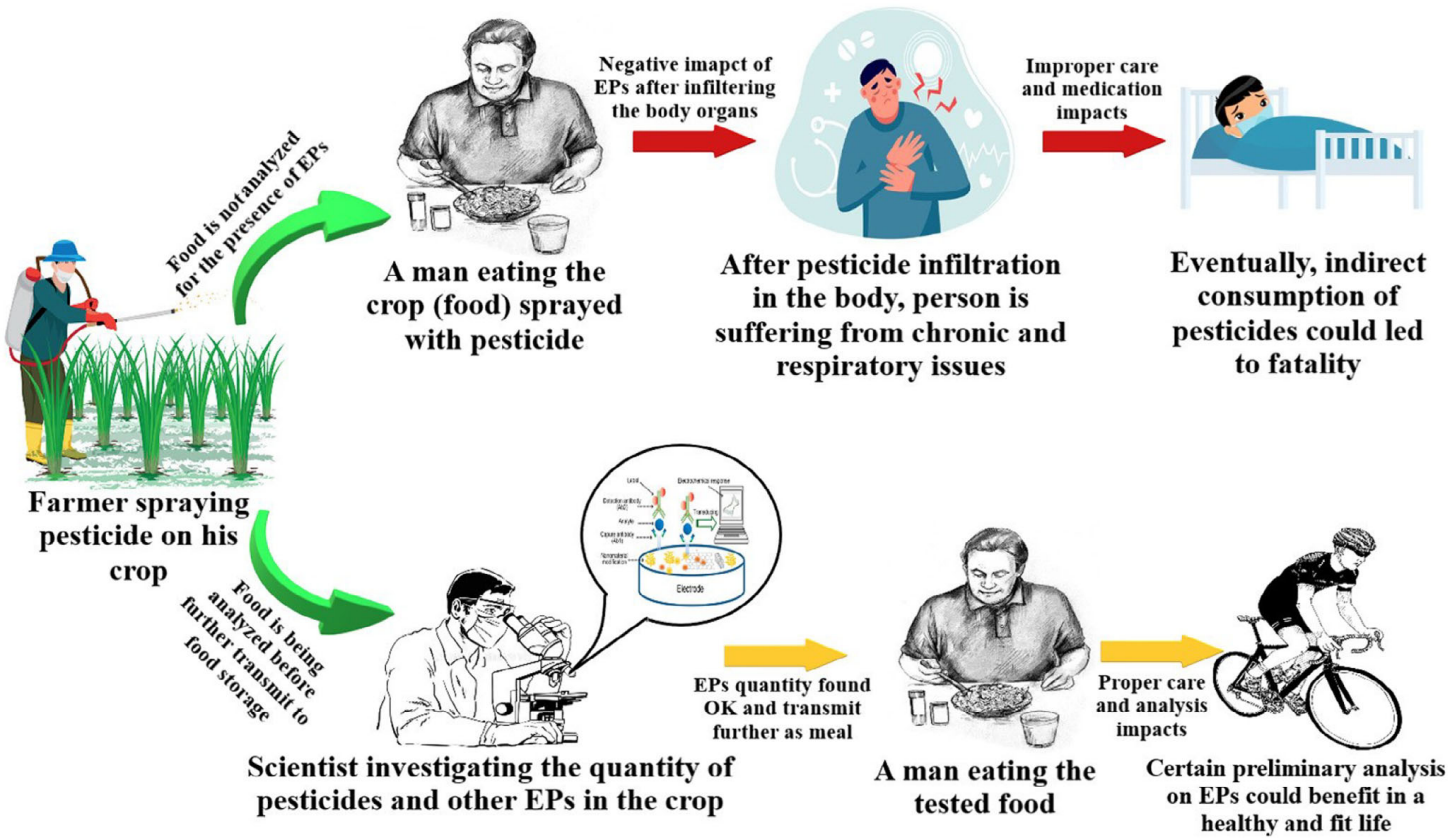
Schematic of rapid detection and remediation of environmental pollutants utilizing nanomaterials‐based biosensors. Reproduced with permission, Copyright 2022, Elsevier.^[^ ^168^ ^]^

The food and beverage industry is a significant and expanding market for sensor technologies, driven by the need for enhanced food safety, quality control, and process optimization.^[^ ^163^ ^]^ Sensors in this industry play a critical role in monitoring and ensuring the safety and quality of food products throughout the supply chain, from production and processing to packaging and distribution. Food safety is a paramount concern, and sensors are increasingly used to detect contaminants, pathogens, and spoilage indicators in real‐time, helping to prevent foodborne illnesses and ensuring compliance with stringent regulatory standards.^[^ ^164^ ^]^ For instance, sensors that monitor temperature, humidity, and gas concentrations are essential in cold chain management, ensuring that perishable goods like dairy, meat, and produce are stored and transported under optimal conditions to preserve freshness and prevent spoilage. In processing plants, sensors are employed to monitor critical parameters such as pH levels, moisture content, and chemical composition, enabling precise control over production processes and ensuring consistent product quality. Additionally, the rise of smart packaging, which integrates sensors to monitor the condition of food products, provides consumers and retailers with real‐time information about the freshness and safety of their purchases, reducing waste and enhancing consumer trust.^[^ ^165^ ^]^ In the context of sustainability, sensors are also being used to optimize resource usage in food production, such as water and energy consumption, contributing to more environmentally friendly practices. The integration of IoT and advanced analytics with sensor data is further transforming the industry, allowing for predictive maintenance, process automation, and real‐time monitoring, which collectively improve efficiency and reduce operational costs. The growing consumer demand for transparency, along with the increasing regulatory requirements for traceability and safety, is driving the adoption of sophisticated sensor technologies in the food and beverage sector.^[^ ^166^ ^]^ As the industry continues to innovate and respond to evolving consumer preferences, the market for sensors is expected to grow, offering significant opportunities for companies that can provide reliable, accurate, and cost‐effective solutions tailored to the specific needs of the food and beverage industry. The need to protect food items from the dangerous effects of infections and toxic microbial metabolites is growing these days due to increased public awareness of the need of eating safe and nutritious food. Due to their capacity to cause foodborne illnesses and intoxications, these organisms and their toxins do, in fact, pose a serious threat to public health and food safety. Common operations are expensive to examine, time‐consuming, labor‐intensive, and require new technologies and trained personnel. Therefore, expanding sensing techniques—such as bio/nanosensors for detections and evaluations, which are rapid, reliable, cost‐effective, and advantageous—is crucial to overcoming such challenges. With the emergence of nanotechnology and artificial intelligence, nanosensors for the detection of microbes and their poisons have been extensively studied.^[^ ^167^ ^]^ **Figure** **15** depicts the steps involved in both remediation and rapid detection of environmental pollutants utilizing nanomaterials‐based biosensors.

#### 4.1.4 Aerospace and Defense

**Figure 16:**
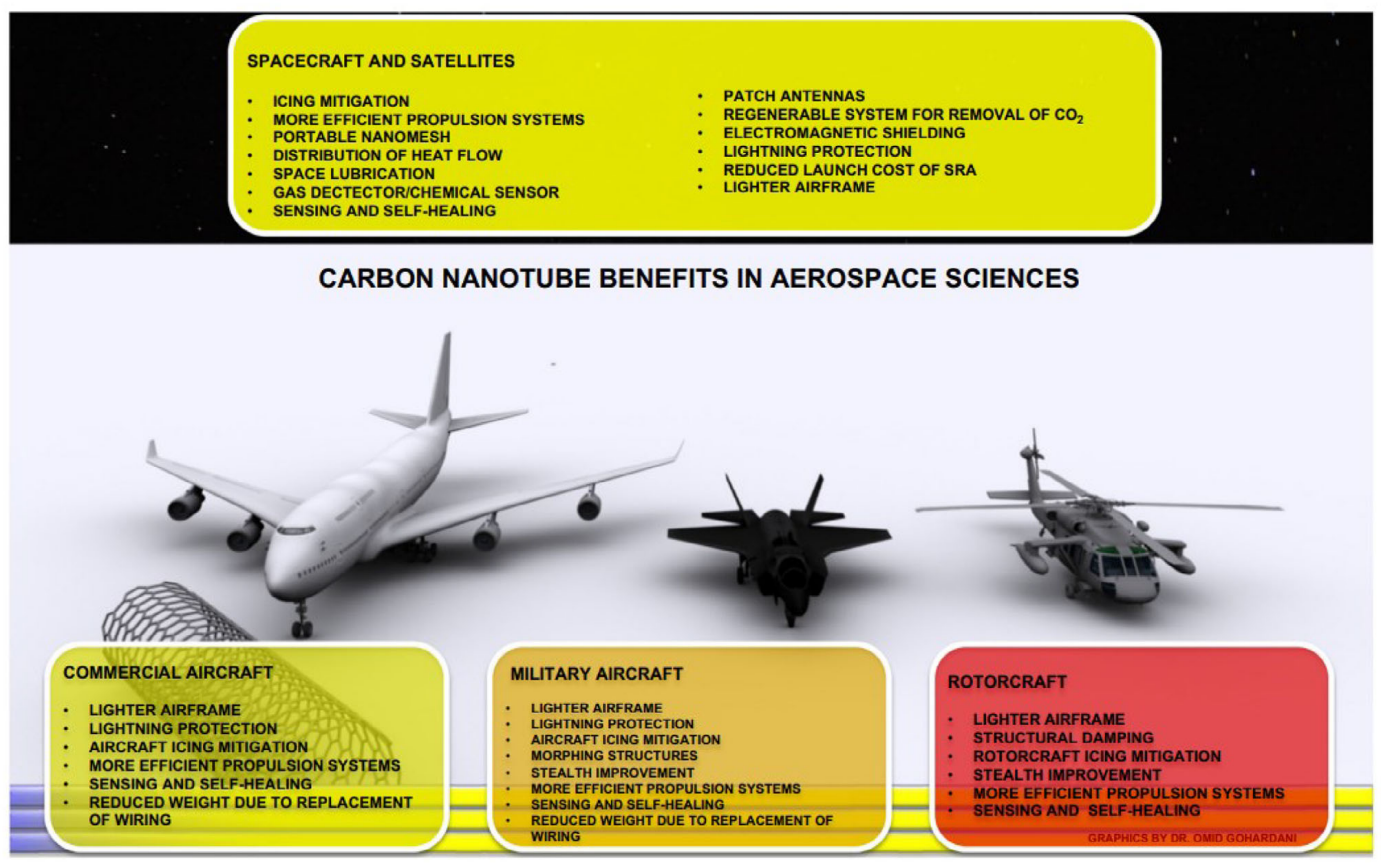
Advantages of applying CNTs in rotorcraft, military aircraft, and commercial aircraft for space applications. Reproduced with permission, Copyright 2014, Elsevier.^[^ ^175^ ^]^

The aerospace and defense market is a highly specialized and critical sector for sensor technologies, driven by the need for precision, reliability, and advanced capabilities in some of the most challenging environments. Sensors in this market are essential for a wide range of applications, including navigation, communication, surveillance, and weapons systems.^[^ ^169^, ^170^ ^]^ In aerospace, sensors are integral to flight control systems, where they monitor parameters such as altitude, airspeed, and orientation, ensuring safe and efficient aircraft operation. They also play a crucial role in avionics, providing data for navigation, collision avoidance, and automated flight systems. The demand for sensors in unmanned aerial vehicles (UAVs) and drones is rapidly growing, as these technologies are increasingly used for reconnaissance, surveillance, and targeted missions. In defense, sensors are pivotal in enhancing situational awareness, detecting threats, and guiding precision weaponry.^[^ ^171^, ^172^, ^173^ ^]^ Advanced radar, infrared, and sonar sensors are used in missile defense systems, surveillance platforms, and combat vehicles to detect and track enemy movements, identify potential targets, and provide critical intelligence in real‐time. The extreme conditions in which these sensors operate—such as high altitudes, varying temperatures, and high G‐forces—require them to be highly durable, reliable, and capable of maintaining accuracy under stress. Additionally, the increasing emphasis on cyber defense and electronic warfare has driven the development of sensors that can detect, analyze, and counteract electronic threats and signal interference. The integration of sensors with artificial intelligence and machine learning is further enhancing their capabilities, enabling faster and more accurate data processing and decision‐making in complex operational environments.^[^ ^174^ ^]^ The aerospace and defense market is characterized by stringent regulatory standards and the need for continuous innovation to maintain technological superiority. As global defense budgets grow and the demand for advanced aerospace systems increases, the market for sensors in this sector is expected to expand, offering significant opportunities for companies that can deliver cutting‐edge, resilient, and highly accurate sensor solutions. Many new uses of carbon nanotubes will be revealed after the commercialization of CNT‐based products in the aerospace sciences, according to the competitiveness of the nanotechnology industry, company intellectual property, and the work of scientists, researchers, and engineers. An overview of the advantages of using CNTs in rotorcraft, space applications, military aircraft, and commercial aviation is given in **Figure** **16**.

#### 4.1.5 Energy and Utilities

The energy and utilities market is a rapidly evolving sector for sensor technologies, driven by the growing need for efficient energy management, grid reliability, and the integration of renewable energy sources.^[^ ^176^, ^177^, ^178^ ^]^ Sensors in this market are crucial for monitoring and optimizing the generation, distribution, and consumption of energy, ensuring that systems operate safely, efficiently, and sustainably. In smart grids, sensors play a vital role in real‐time monitoring of power flow, voltage levels, and equipment health, enabling utilities to detect and respond to outages, manage load distribution, and reduce energy losses.^[^ ^179^ ^]^ These sensors are also essential for predictive maintenance, allowing utility companies to anticipate equipment failures and perform maintenance before issues lead to costly downtimes. The shift towards renewable energy sources such as solar, wind, and hydropower has further increased the demand for advanced sensor technologies.^[^ ^180^ ^]^ For instance, sensors in solar panels and wind turbines monitor environmental conditions and system performance to maximize energy production and improve the longevity of the equipment. Additionally, in oil and gas operations, sensors are used to monitor pipelines, detect leaks, and ensure the safe extraction and transportation of hydrocarbons. The advent of smart meters, which rely on sensors to provide detailed, real‐time data on energy usage, has empowered consumers and businesses to better manage their energy consumption, contributing to more efficient and sustainable energy use. Moreover, the integration of sensors with IoT platforms and big data analytics is transforming the energy sector by enabling more sophisticated energy management systems that can predict demand, optimize energy storage, and enhance grid resilience.^[^ ^181^ ^]^ As global energy demand continues to rise and the push for decarbonization intensifies, the need for innovative sensor technologies in the energy and utilities market is expected to grow significantly. This presents substantial opportunities for companies that can develop reliable, accurate, and scalable sensor solutions tailored to the unique challenges of the energy industry.

#### 4.1.6 Consumer Electronics

The consumer electronics market is one of the largest and most dynamic sectors for sensor technologies, driven by the rapid pace of innovation and the growing demand for smart, connected devices. Sensors are integral to a wide range of consumer electronics, from smartphones and wearables to home automation systems and gaming consoles.^[^ ^182^ ^]^ In smartphones, sensors such as accelerometers, gyroscopes, proximity sensors, and biometric sensors enable features like motion detection, facial recognition, and augmented reality, enhancing user experience and device functionality.^[^ ^183^, ^184^, ^185^ ^]^ The rise of wearable technology, including fitness trackers and smartwatches, relies heavily on sensors to monitor physical activity, heart rate, sleep patterns, and other health metrics, empowering users to take control of their health and wellness. In the home, sensors are central to the development of smart home devices, including security systems, thermostats, lighting controls, and voice‐activated assistants, which offer increased convenience, energy efficiency, and security. The gaming industry also leverages sensors, particularly in virtual reality (VR) and augmented reality (AR) systems, where motion tracking and spatial awareness are key to creating immersive experiences. The continuous miniaturization and improvement in sensor accuracy and power efficiency are enabling more sophisticated applications and driving the proliferation of Internet of Things (IoT) devices, which connect and interact seamlessly in smart ecosystems. Additionally, the integration of artificial intelligence (AI) with sensor technology is opening new possibilities for personalized, context‐aware interactions in consumer electronics. The demand for sensors in consumer electronics is further fueled by the global trend towards digitization and the growing expectations for high‐performance, multifunctional devices.^[^ ^186^ ^]^ As consumers increasingly seek devices that are not only smart but also intuitive and responsive to their needs, the market for sensors in consumer electronics is set to expand, presenting significant opportunities for companies that can innovate and deliver cutting‐edge sensor solutions that enhance user experience and drive the next generation of smart devices. The function of electrochemical biosensors in SARS‐CoV‐2 detection was originally explained by Mao et al.^[^ ^187^ ^]^ using bibliometric analysis. The contributions made to this subject by various nations and organizations were examined. Several methodological approaches at electrochemical detection methods were investigated using keyword analysis.

### 4.2 Assess Market Demand

**Figure 17:**
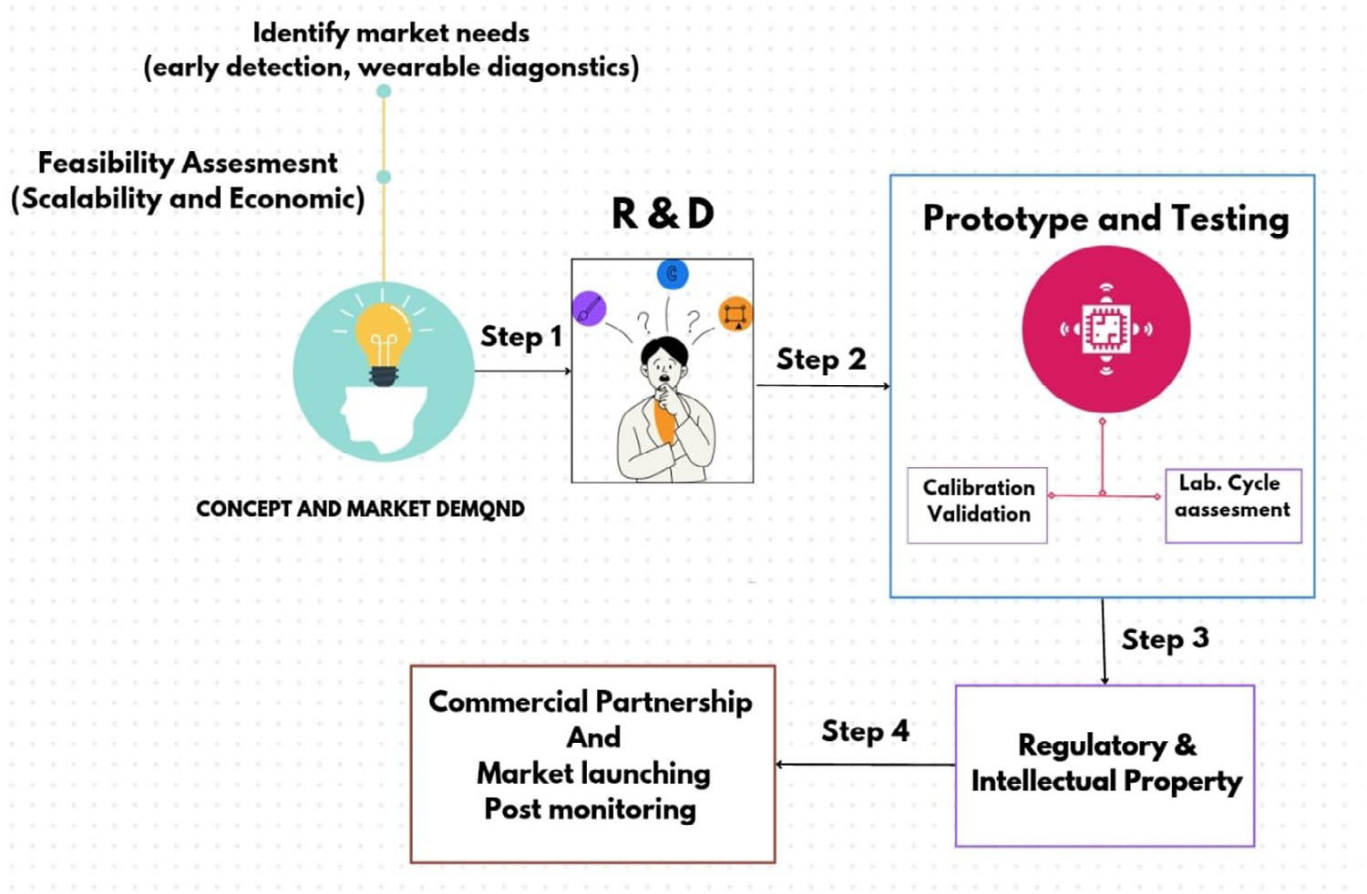
The proper roadmap of the nanomaterials‐based sensors for the commercialization process.

Assessing market demand for sensors involves a comprehensive analysis of various factors that drive the adoption and growth of sensor technologies across different industries. Market demand for sensors is influenced by technological advancements, regulatory requirements, consumer preferences, and the broader trends in digitization and automation. As sensors become more sophisticated, miniaturized, and cost‐effective, their application across sectors has expanded, leading to increased market demand. To assess this demand, it is crucial to examine key industries that are the primary consumers of sensor technologies, such as healthcare, automotive, consumer electronics, industrial automation, energy, and environmental monitoring. Globally, the market for nanosensors has grown significantly in terms of both finances and the economy. According to recent estimations, its worth ranges from $637 million to $700 million. Due to the growing need for nanosensors in the chemical, medicinal, ecological, and electronics sectors, analysts predict that the nanosensor business will yield an average return on investment of 7% to 11%. The market is anticipated to be worth between $2.37 billion and $3.1 billion by 2032.^[^ ^188^, ^189^ ^]^ In healthcare, the demand for sensors is growing rapidly due to the increasing focus on personalized medicine, preventive healthcare, and the aging global population. Wearable devices, remote patient monitoring systems, and diagnostic tools all rely heavily on sensors to provide accurate, real‐time health data. This trend is further driven by the rising prevalence of chronic diseases, which necessitates continuous monitoring and management. The COVID‐19 pandemic has also accelerated the adoption of telemedicine and home healthcare solutions, significantly boosting the demand for sensors in medical devices. The automotive industry is another significant driver of sensor demand, particularly with the ongoing development of advanced driver‐assistance systems (ADAS), electric vehicles (EVs), and autonomous vehicles (AVs). Sensors such as lidar, radar, cameras, and ultrasonic sensors are critical for ensuring the safety, reliability, and efficiency of these technologies. As automakers continue to innovate and integrate more autonomous features, the demand for sensors in the automotive sector is expected to grow exponentially. In the consumer electronics market, the proliferation of smart devices, including smartphones, wearables, and smart home products, has led to a surge in demand for sensors. Consumers increasingly expect their devices to be intuitive, responsive, and capable of providing a seamless user experience. Sensors such as accelerometers, gyroscopes, proximity sensors, and biometric sensors are essential in meeting these expectations. Additionally, the rise of virtual reality (VR) and augmented reality (AR) technologies, which rely heavily on advanced motion and environmental sensing, further fuels the demand for sensors in this sector. Industrial automation and the broader trend toward Industry 4.0 are also major contributors to the growing demand for sensors. Factories and production facilities are increasingly adopting automation and smart manufacturing processes, which require a wide array of sensors to monitor and control various aspects of production, including temperature, pressure, proximity, and machine health. Predictive maintenance, which relies on sensors to detect early signs of equipment failure, is another key area driving sensor demand in industrial settings. The integration of sensors with IoT platforms allows for real‐time data collection and analysis, enabling more efficient and responsive manufacturing processes. The energy and utilities sector is experiencing growing demand for sensors as well, particularly with the shift toward renewable energy sources and the development of smart grids. Sensors are used to monitor and optimize the generation, distribution, and consumption of energy, ensuring the efficiency and reliability of energy systems. In renewable energy, sensors play a crucial role in maximizing the performance of solar panels, wind turbines, and other renewable energy installations. The push for decarbonization and the need for better energy management solutions are expected to continue driving demand for sensors in this sector. Environmental monitoring is another key area where sensor demand is on the rise. As concerns about climate change, pollution, and resource management grow, the need for accurate and reliable environmental data has become more pressing. Sensors used in air and water quality monitoring, weather stations, and soil analysis provide critical information for governments, industries, and environmental organizations to make informed decisions and comply with regulations. The increasing severity of environmental challenges, such as extreme weather events and pollution, is likely to drive further demand for sensors in this field. Assessing market demand for sensors also involves understanding the impact of regulatory and safety standards across various industries. In sectors like healthcare, automotive, and aerospace, strict regulatory requirements necessitate the use of high‐precision, reliable sensors to ensure compliance with safety and quality standards. As regulations continue to evolve and become more stringent, the demand for advanced sensor technologies that meet these requirements is likely to increase. Moreover, global economic trends and geopolitical factors can influence sensor demand. For instance, the ongoing digital transformation and the rise of smart cities, coupled with government initiatives to promote technological innovation, are likely to boost sensor adoption. On the other hand, supply chain disruptions, trade tensions, and economic uncertainties can impact the availability and cost of sensors, affecting market demand. In conclusion, assessing market demand for sensors requires a multifaceted approach that considers the technological, regulatory, and economic factors driving sensor adoption across various industries. As sensors become increasingly integral to the functioning of modern technologies and systems, their demand is expected to grow across multiple sectors. Companies that can innovate and offer reliable, cost‐effective, and high‐performance sensor solutions are well‐positioned to capitalize on this expanding market. Understanding the specific needs and trends within each industry is essential for accurately forecasting sensor demand and identifying the most promising opportunities for growth and investment. **Figure** **17** shows the proper roadmap of the nanomaterials‐based sensors for the commercialization process. This roadmap describes the key steps from lab‐scale research to market entry, identifies the major challenges and opportunities, prototype and testing, regulatory and intellectual property, etc.

### 4.3 Feasibility Study

A feasibility study for a sensor involves a thorough analysis of the technical, operational, financial, and market aspects to determine the viability of developing and deploying the sensor technology. The objective of such a study is to assess whether the sensor can be successfully brought to market, considering various factors such as technical feasibility, cost‐effectiveness, market demand, regulatory compliance, and potential return on investment (ROI). The process typically begins with a clear understanding of the sensor's intended application, the problem it aims to solve, and the specific requirements of the target industry. This foundational step is crucial as it shapes the subsequent evaluation of the sensor's design, functionality, and performance metrics. Technical feasibility is one of the primary considerations in the study. This involves evaluating the sensor's design and engineering challenges, including the choice of materials, power consumption, size, sensitivity, accuracy, and durability. The study must assess whether the current state of technology can support the development of the sensor with the desired specifications. For instance, if the sensor is intended for a high‐precision application, the feasibility study would examine the availability of technologies that can achieve the required levels of accuracy and reliability. Additionally, the study should consider the manufacturing processes required to produce the sensor at scale, ensuring that the necessary infrastructure and expertise are available. Prototyping and testing are critical components of the technical feasibility analysis, as they provide tangible insights into the sensor's performance under real‐world conditions. This stage may also involve identifying potential technical risks, such as issues with miniaturization, signal processing, or data integration, and developing strategies to mitigate these risks. Operational feasibility involves examining the practicality of integrating the sensor into existing systems and processes. This includes assessing the ease of installation, compatibility with other devices or platforms, and the level of maintenance required. For example, in an industrial setting, the sensor must be compatible with existing automation systems and capable of withstanding harsh operating conditions, such as extreme temperatures, vibrations, or corrosive environments. The study should also evaluate the training requirements for personnel who will operate or maintain the sensor, as well as the support infrastructure needed, such as software for data analysis and interpretation. Operational feasibility also considers the logistics of production, including the supply chain for components, manufacturing timelines, and potential bottlenecks that could impact the sensor's deployment. Ensuring that the sensor can be seamlessly integrated and maintained without disrupting operations is crucial for its success. Financial feasibility is another critical aspect of the study, focusing on the cost‐effectiveness of developing and commercializing the sensor. This involves estimating the total cost of development, including research and development (R&D), prototyping, testing, regulatory approvals, and manufacturing. The study should also consider the costs associated with marketing, distribution, and ongoing support. A detailed financial analysis would compare these costs against the potential revenue generated from sales, licensing, or other revenue streams. This analysis helps determine whether the project is financially viable and if it can achieve a satisfactory ROI. Additionally, the study should consider funding options, such as venture capital, government grants, or partnerships, that could support the development and commercialization of the sensor. Sensitivity analysis, which examines how changes in key assumptions (such as production costs or market demand) affect the financial outcomes, is also an important tool in assessing financial feasibility. Market feasibility involves analyzing the potential demand for the sensor, identifying target customers, and assessing the competitive landscape. This requires a deep understanding of market trends, customer needs, and the positioning of the sensor relative to existing products. The study should evaluate the size and growth rate of the target market, as well as the willingness of customers to adopt the new technology. Understanding the competitive landscape is crucial, as it helps identify differentiating factors that can give the sensor a competitive edge, such as superior performance, lower cost, or unique features. The study should also explore potential barriers to market entry, such as regulatory hurdles, intellectual property issues, or entrenched competitors, and develop strategies to overcome these challenges. Marketing strategies, including pricing, distribution channels, and promotional activities, should be outlined to ensure the sensor reaches its intended audience effectively. Regulatory and legal feasibility is another important consideration, particularly in industries such as healthcare, automotive, or aerospace, where stringent regulations govern product development and deployment. The feasibility study should identify relevant regulations and standards that the sensor must comply with, such as safety certifications, environmental regulations, or data privacy laws. Navigating the regulatory landscape can be complex and time‐consuming, so the study should assess the time and cost implications of obtaining the necessary approvals and certifications. Intellectual property (IP) considerations, such as patents or trademarks, should also be addressed to protect the sensor's technology and avoid potential legal disputes. In conclusion, a feasibility study for a sensor is a comprehensive evaluation process that addresses technical, operational, financial, market, and regulatory aspects to determine the viability of developing and commercializing the sensor technology. By thoroughly assessing these factors, the study provides valuable insights into the risks and opportunities associated with the project, helping stakeholders make informed decisions about whether to proceed with development. A successful feasibility study not only ensures that the sensor can be developed and deployed effectively but also sets the stage for its long‐term success in the market, maximizing its impact and profitability.

## 5 Technology Transfer and Commercialization Process

**Figure 18:**
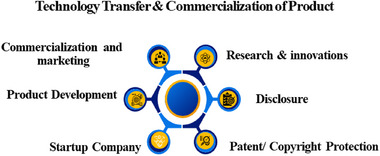
Steps involved in technology transfer and commercialization process.

The process of bringing a revolutionary technology from the laboratory to the market is comprised of a number of consecutive phases (**Figure** **18**). When it comes to the creation of one‐of‐a‐kind technologies, the development of those technologies for use in industry, or the commercialization of the goods it produces, it is essential to have a solid understanding of the innovation life cycle.

### 5.1 Research Innovation

Inventions are often the result of observations and experiments that take place during research operations. For a number of different reasons, it is essential to maintain meticulous and trustworthy laboratory records of research operations. Please do not forget to get in touch with the Technology Transfer Office before transmitting any research materials to locations outside of the University. Before disseminating the findings of any study or publishing what has been discovered, it is essential to enquire with the Technology Transfer Office as to whether the document or disclosure includes any innovations or discoveries that might be protected by a patent.

### 5.2 Pre‐Disclosure

The inventor needs to get in touch with the Trademark Office (TTO) as soon as possible to discuss the innovation in order to ascertain whether or not the timing is suitable to submit an invention disclosure form. Multiple researchers often contribute to an innovation, and defining who the inventor is is essential due to the fact that the law stipulates that the only people who are considered to be inventors are those who have produced independent, intellectual contributions to an invention. At this stage, inventors may also be of assistance by conducting a comprehensive search for previous art.

### 5.3 Invention Disclosure

A Form for Disclosure of Invention will be filled out by the inventor and then sent to the Trademark Office. It is with this written disclosure that the official process of technology transfer gets underway. An invention disclosure is a document that is kept secret and should include a comprehensive description of your innovation. This is done so that the various commercialization alternatives may be assessed and evaluated further.

### 5.4 Assessment

Announcement of an Invention Forms that are received by the TTO are recorded and checked for accuracy. A presentation before a patent committee that lasts for fifteen minutes is something that the inventor is obligated to provide. Evaluation of the technology is carried out with the participation of the inventor. In order to determine whether or not the innovation has the potential to be commercialized, any patent searches that are required are accomplished, and market research is carried out. In order to determine whether the strategy should be centered on licensing to an existing firm or on assisting the inventor in establishing a new start‐up business, the assessment process will serve as a guide.

### 5.5 Patent and Copyright Protection

Depending on the results of the evaluation, the authority in charge will decide whether or not to submit a patent application for the innovation. As a result of the high cost of filing, patent protection is not sought for all invention disclosures that are received. Outside patent lawyers are responsible for the filing of patent applications as well as their subsequent prosecution. The patent attorney will have some familiarity with the invention's area, but it is very improbable that they will be an expert in the subject. Therefore, it is very necessary for the inventor to participate in the timely examination and preparation of the patent application before it is submitted. It is the responsibility of the inventor to furnish the attorney with any material that may be necessary by the attorney in order to acquire effective patent protection. This includes any details that make the invention new, useful, and non‐obvious that the attorney may demand.

### 5.6 Licensing

The respective office is responsible for draughting individualized contracts between the University and all parties involved. Licensing allows one to get the rights to an innovation without giving up ownership of the idea. Through the use of an exclusive license, the University will guarantee that it will keep the right to use and manufacture the invention for research purposes, allowing the inventor to continue his or her study on the subject. On the other hand, the process of locating a suitable licensee might take several months or even years, depending on the attractiveness of the innovation as well as the size of the market and the stage of development it is now in. As an example, the process of obtaining a license for a research material may take one week, but the process of acquiring a portfolio of pharmaceutical compounds could take one year. When licensing technology, there are often two different licensing pathways that may be taken.

#### 5.6.1 Traditional License

A license for the innovation is granted to a third party by a representative of the Trademark Office. If the University is successful in licensing the idea to a third party, the institution will divide any money that is received from the license in line with the policy regarding patents or copyrights. Due to the fact that the majority of university innovations are often in the early stages of the development cycle and need significant investments for commercialization, it might be challenging to attract many licensees. Following the identification of a licensee, the process of negotiating particular conditions for various items will take varying periods of time.

#### 5.6.2 Start‐Up Company

If the innovation is a platform technology and the inventor is interested in starting a start‐up firm, a representative from the TTO will explain the regulations and conditions that are required to get a license from the University. The Research and Sponsored Programs Office of the University will create two conflict‐of‐interest papers after the Dean and Chair have reached a consensus that it is OK for the faculty member to continue. These documents will be divided into two categories: 1) Between the faculty member and the University, and 2) Between the startup firm and the University. Once the documentation pertaining to conflicts of interest have been signed, a representative from the Trade and Technology Office will start the process of negotiating the license agreement. Please be aware that the University inventor is not authorized to take part in this procedure. Instead, the University inventor is strongly recommended to seek the advice of legal counsel in order to represent the interests of the newly created firm. The following is a list of the financial conditions that are often included in a license for a university start‐up establishment: One (1) license fee paid up front; two (2) royalty rates that are common in the industry; three (3) minimum yearly royalty; four (4) sublicense fees; five (5) milestone payments; six (6) a percentage of equity; and seven (7) reimbursement of all patent expenditures.

### 5.7 License Management

Maintenance and cataloging of the license will be performed by the Technology Transfer Office during the duration of the patent. In order to guarantee recovery for patenting expenses and payments that are required in accordance with the license, the performance of a license will be examined and checked on a quarterly basis.

### 5.8 Product Development

Designing, engineering, and testing a product are the primary components of the product development process. In order to find and apply for Small Business Innovation Research (SBIR) and Small Business Technology Research (STTR) grants, university academics and start‐ups may collaborate with the Office of Research and Sponsored Programs, Rocket Innovations, and Business Incubator to pay the costs involved with the development of a product. It will also be necessary for the start‐up to do market research and market analysis in order to decide the strategy that the firm intends to use in order to join the market.

### 5.9 Commercialization and Marketing

**Figure 19:**
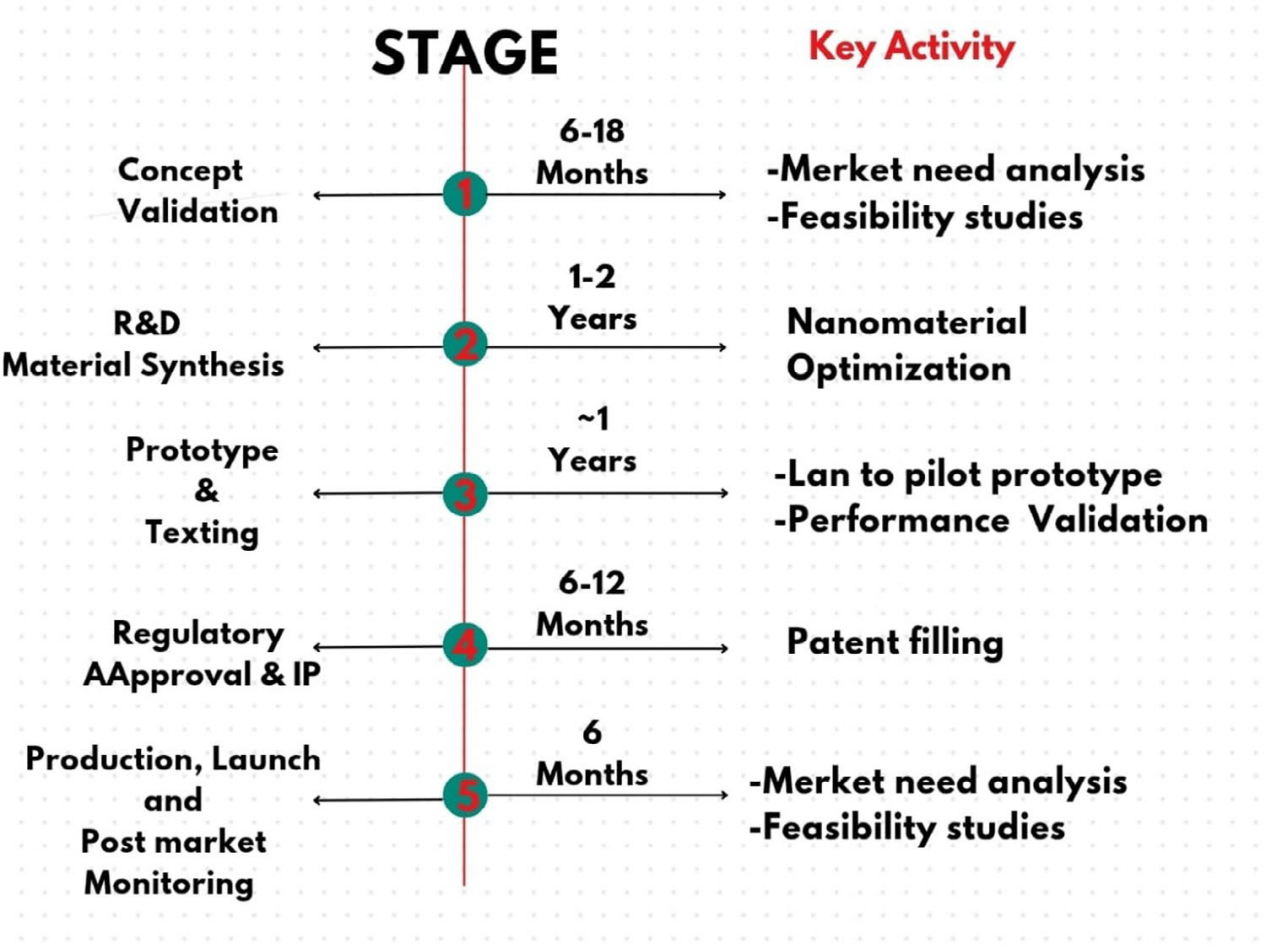
Timeline diagram with average duration for each stage.

The firm that has the license is responsible for continuing the development of the technology and making further financial expenditures in order to further develop the product or service. It is possible that this stage will include more development, authorization from regulatory agencies, sales and marketing, support, training, and other operations. The TTO will perform market research with the participation of the inventor in order to identify potential organizations that possess the skills, resources, and business networks necessary to render the innovation ready for commercialization. An active participation on the part of the inventor in the process of producing material to be given to corporations has the potential to significantly increase the success of this procedure. During the marketing stage, it is possible to make use of both non‐confidential summaries of the technology as well as confidential information that is protected by a confidentiality agreement. The timeline diagram with average duration for each step is depicted in **Figure** **19**.

## 6 Challenges

**Figure 20:**
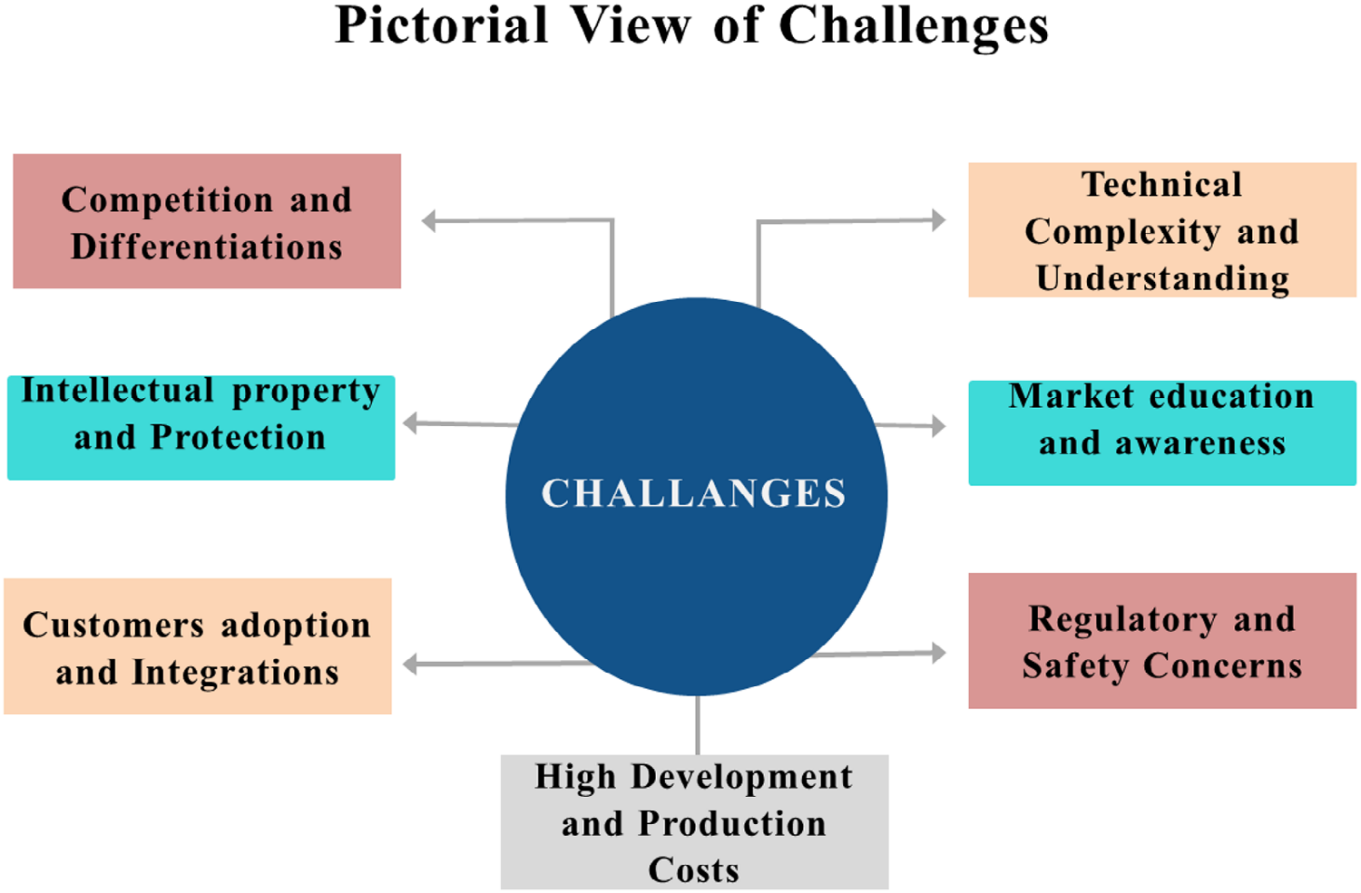
Pictorial view of challenges.

Marketing strategies for nanomaterials in sensor applications face several unique challenges (**Figure** **20**) that can impact their success in the market.

### 6.1 Technical Complexity and Understanding

Technical complexity and understanding represent significant challenges in marketing nanomaterials for sensor applications due to the intricate nature of the technology and its scientific underpinnings. Nanomaterials, with their advanced properties and applications, involve concepts such as quantum effects, nanoscale interactions, and specialized fabrication techniques that can be difficult for non‐experts to grasp. Effectively communicating these complex details to a broad audience requires simplifying technical jargon while accurately conveying the technology's benefits. This challenge is compounded by the need to educate potential customers about how nanomaterials improve sensor performance compared to conventional materials. Developing marketing strategies that make complex scientific information accessible involves creating clear, engaging content such as infographics, case studies, and demonstrations that highlight practical applications and real‐world advantages. Additionally, staying abreast of rapid advancements in nanotechnology is essential to ensure that marketing messages remain current and relevant. Leveraging expert endorsements and collaborating with industry influencers can also enhance credibility and help bridge the knowledge gap. By addressing the technical complexity with a focus on clarity and education, marketers can more effectively convey the value of nanomaterial‐based sensors and drive adoption in the market.

### 6.2 Market Education and Awareness

Market education and awareness pose significant challenges in marketing nanomaterials for sensor applications due to the relatively novel and evolving nature of the technology. As nanomaterials are still emerging in many industries, there is often limited understanding among potential customers about their benefits and applications. This lack of familiarity requires marketers to invest considerable effort in educating the market, which involves not only explaining the technical advantages of nanomaterials but also demonstrating their practical impact on sensor performance. Effective market education strategies must include the creation of informative content such as white papers, technical articles, webinars, and interactive demonstrations that clearly articulate the value proposition of nanomaterials. Engaging in direct outreach to industry stakeholders through workshops, trade shows, and conferences can also help raise awareness and build trust. Furthermore, establishing thought leadership and collaborating with academic institutions and industry experts can enhance credibility and facilitate deeper understanding. Overcoming the challenge of market education requires a strategic approach that combines clear communication, practical demonstrations, and ongoing engagement to bridge the knowledge gap and drive the adoption of nanomaterial‐based sensors.

### 6.3 Regulatory and Safety Concerns

Regulatory and safety concerns represent significant challenges in marketing nanomaterials for sensor applications due to stringent and often complex regulations governing their use. Nanomaterials are subject to rigorous scrutiny because of potential health, safety, and environmental impacts, which vary across different regions and industries. Navigating these regulations requires a thorough understanding of the applicable standards and a proactive approach to ensure compliance. Marketers must address these concerns by transparently communicating the safety measures and regulatory certifications associated with their nanomaterial‐based sensors. This includes providing detailed information on the testing and validation processes that demonstrate the materials' safety and environmental impact. Additionally, securing relevant approvals and certifications can be time‐consuming and costly, adding to the complexity of bringing the technology to market. To mitigate these challenges, effective marketing strategies should include clear messaging about the adherence to regulatory standards, the steps taken to ensure safety, and any third‐party endorsements or certifications. Engaging with regulatory experts and incorporating their insights into the marketing strategy can also help address potential concerns and build trust with stakeholders. Overall, managing regulatory and safety challenges requires a transparent and informed approach to ensure that the technology is perceived as both compliant and reliable in the market.^[^ ^190^ ^]^

### 6.4 High Development and Production Costs

High development and production costs are a significant challenge in marketing nanomaterials for sensor applications, primarily due to the specialized processes and advanced technologies required for their creation. Nanomaterials often involve intricate synthesis techniques and precise manufacturing conditions that can drive up expenses compared to traditional materials. These high costs can impact the pricing strategy and affordability of the final sensor products, potentially limiting their market appeal, especially in price‐sensitive sectors. Marketing strategies must address these financial challenges by clearly communicating the long‐term value and performance benefits of nanomaterial‐based sensors, such as enhanced accuracy, durability, and functionality, which justify the higher initial investment.^[^ ^191^ ^]^ Highlighting partnerships, funding opportunities, or cost‐sharing models can also make the technology more accessible. By effectively conveying the value proposition and addressing cost concerns, marketers can help mitigate the impact of high development and production costs and drive adoption of nanomaterial‐based sensors.

### 6.5 Competition and Differentiation

Competition and differentiation are critical challenges in marketing nanomaterials for sensor applications, given the highly competitive landscape and the presence of numerous alternative technologies. In a market saturated with diverse sensor solutions, standing out requires clearly articulating the unique advantages and superior performance of nanomaterial‐based sensors. This involves not only highlighting the specific benefits of nanomaterials, such as enhanced sensitivity, miniaturization, or multifunctionality, but also differentiating these sensors from both traditional and other advanced technologies. Effective marketing strategies must focus on building a strong value proposition that emphasizes how the unique properties of nanomaterials address specific customer needs or solve problems better than competing solutions. Additionally, showcasing success stories, case studies, and real‐world applications can provide tangible evidence of the technology's benefits and help establish credibility. Developing a strong brand identity and leveraging intellectual property, such as patents, can further reinforce differentiation. To navigate this competitive environment, marketers must continuously monitor industry trends, competitor activities, and emerging technologies to adapt their strategies and maintain a competitive edge.^[^ ^192^ ^]^

### 6.6 Intellectual Property Protection

Intellectual property (IP) protection poses a significant challenge in marketing nanomaterials for sensor applications, given the high value of innovation in this rapidly evolving field. Securing patents and protecting proprietary technologies are crucial for maintaining a competitive advantage and ensuring that novel nanomaterial‐based sensors are not easily replicated by competitors. However, navigating the complexities of patent law, securing global IP rights, and managing IP portfolios can be both costly and time‐consuming. Marketing strategies must address these concerns by clearly communicating the technological uniqueness and proprietary aspects of the sensor products to differentiate them from competitors. This involves not only highlighting patented technologies and innovations but also demonstrating the strategic steps taken to protect and defend these intellectual assets.^[^ ^193^ ^]^ Engaging with legal experts to navigate IP challenges and integrating IP management into the broader business strategy are essential for successfully marketing nanomaterial‐based sensors and leveraging their technological advantages in the market.

### 6.7 Customer Adoption and Integration

Customer adoption and integration present significant challenges in marketing nanomaterials for sensor applications, as the transition from traditional technologies to advanced nanomaterial‐based sensors can be complex and daunting for many organizations. Potential customers may be hesitant to adopt new technologies due to concerns about compatibility with existing systems, the complexity of integration, or the learning curve associated with new products. Effective marketing strategies must address these concerns by demonstrating the ease of integration and providing comprehensive support throughout the adoption process. This includes offering detailed technical documentation, integration guides, and robust customer support to facilitate a smooth transition. Additionally, showcasing successful case studies and pilot programs can help build confidence by illustrating real‐world applications and the benefits realized by early adopters. Engaging with potential customers through hands‐on demonstrations, webinars, and trials can also help alleviate concerns and provide a tangible understanding of the technology's value. By addressing adoption and integration challenges proactively and offering tailored support, marketers can encourage broader acceptance and successful implementation of nanomaterial‐based sensors in various applications.^[^ ^194^ ^]^

Nanomaterials hold immense promise for revolutionizing sensor technology, offering enhanced sensitivity, selectivity, and responsiveness. However, translating these lab‐proven advantages into commercially successful products requires a robust marketing strategy that addresses the unique challenges and opportunities presented by this cutting‐edge technology. The inherent complexity of nanomaterials and their sensor applications presents a significant marketing challenge. Nanomaterials themselves possess unique properties and behaviors that require specialized knowledge to understand, making it difficult to explain their functionality and benefits to a broader audience. Furthermore, the integration of nanomaterials into sensor devices and their application in specific industries often involves intricate technical details that can be overwhelming for potential customers. This complexity necessitates clear, concise, and accessible communication strategies that break down complex concepts into digestible information, avoiding technical jargon and focusing on the tangible benefits and value proposition for the end‐user. Effectively addressing this complexity is crucial for building customer understanding, trust, and ultimately, driving market adoption. Trust and safety concerns surrounding nanomaterials are also a challenge to their successful commercialization, particularly in sensor applications. Public perception can be negatively influenced by a lack of understanding about nanotechnology and anxieties about potential health and environmental risks. This necessitates proactive and transparent communication from companies regarding the specific nanomaterials used in their sensors, rigorous safety testing protocols, and clear explanations of how these materials are handled and contained within the devices. Building trust is crucial and can be achieved through independent validation of safety data, collaboration with reputable organizations, and open dialogue with stakeholders to address concerns and foster confidence in the responsible use of nanomaterials. High production costs arise a hurdle in the commercialization of nanomaterial‐based sensors. The synthesis and processing of high‐quality nanomaterials often involve complex and energy‐intensive procedures, requiring specialized equipment and expertise. This translates to substantial manufacturing expenses, which can limit the affordability and widespread adoption of these sensors. Furthermore, scaling up production while maintaining consistent quality and performance can be challenging, further contributing to the high costs. Overcoming this challenge requires continuous innovation in manufacturing processes, exploring cost‐effective synthesis methods, and optimizing production workflows to reduce expenses and make nanomaterial‐based sensors more accessible to a broader market. The advanced sensor market is highly competitive, featuring both established companies with extensive experience and resources, and agile startups introducing innovative technologies. This competitive landscape presents a significant challenge for nanomaterial‐based sensors, requiring them to demonstrate clear advantages over existing solutions in terms of performance, cost, or unique functionalities.^[^ ^195^ ^]^ Successfully navigating this competition demands a well‐defined value proposition, a robust marketing strategy, and potentially strategic partnerships to gain market share and establish a strong foothold.

Some solutions to the challenges associated with marketing nanomaterials for sensor applications effective targeted communication is crucial for marketing nanomaterial‐based sensors. This involves clearly articulating the specific benefits and advantages these sensors offer over existing solutions, focusing on their superior performance, enhanced sensitivity, and unique capabilities. Simplifying complex technical concepts into easily understandable language, avoiding jargon, and using visuals like diagrams, charts, and real‐world case studies are essential. Furthermore, tailoring the communication message to resonate with specific target audiences, and addressing their individual needs and concerns ensures that the value proposition is clearly understood and appreciated, driving interest and adoption. Building trust and credibility is paramount for the successful commercialization of nanomaterial‐based sensors. This involves prioritizing transparency by openly addressing any potential safety concerns and providing clear, accessible information about the nanomaterials used their properties, and their potential impact. Rigorous scientific validation of sensor performance through independent studies, certifications, and real‐world testing is crucial to demonstrate reliability and accuracy. Furthermore, fostering strategic partnerships with established industry leaders, reputable research institutions, and relevant regulatory bodies can significantly enhance credibility and build confidence among potential customers. Open communication, ethical practices, and a commitment to safety are essential for establishing long‐term trust in nanomaterial‐based sensor technology. Strategic marketing and sales are crucial for successfully commercializing nanomaterial‐based sensors. This involves identifying specific target market segments where the unique advantages of these sensors provide a clear competitive edge. Developing a strong brand identity that conveys innovation, quality, and reliability is essential for building customer trust and recognition. Leveraging digital marketing channels, including targeted online advertising, content marketing, and social media engagement, can effectively reach potential customers and generate leads. Building a knowledgeable and well‐trained sales force capable of clearly communicating the value proposition and addressing technical inquiries is also vital. A comprehensive marketing and sales strategy should integrate these elements to effectively position nanomaterial‐based sensors in the market and drive sales growth. Addressing the cost and scalability of nanomaterial‐based sensors is crucial for successful commercialization. Optimizing production processes is essential to reducing the high costs associated with manufacturing high‐quality nanomaterials. This involves streamlining synthesis methods, improving yields, and minimizing waste. Exploring strategic partnerships with established manufacturers and distributors can help achieve economies of scale, lowering per‐unit costs and expanding market reach. Implementing a value‐based pricing strategy, which reflects the superior performance and benefits offered by these sensors, can justify a potentially higher price point compared to conventional alternatives.^[^ ^196^ ^]^ Continuous improvement in manufacturing techniques and strategic collaborations to achieve cost‐effectiveness and ensure sufficient production capacity to meet market demand.

## 7 Future Scope

The future scope of marketing strategies in nanomaterials for sensor applications is poised to undergo a profound transformation, driven by advancements in technology, evolving market demands, and increasing emphasis on sustainability. As nanotechnology progresses, marketing strategies will need to highlight the cutting‐edge innovations and unparalleled benefits that nanomaterials bring to sensor applications. These include enhanced sensitivity, greater accuracy, and miniaturization capabilities that traditional materials cannot match. Emphasizing these advancements will be crucial in differentiating nanomaterial‐based sensors from existing solutions and demonstrating their superior performance. As a result, marketing efforts will increasingly leverage advanced digital tools and data analytics to create highly targeted campaigns. Artificial intelligence (AI) and machine learning will play a significant role in refining customer segmentation and personalization, enabling marketers to address specific needs and pain points with greater precision. This will enhance the effectiveness of marketing campaigns, driving better engagement and conversion rates. The growing focus on sustainability and environmental impact will also significantly influence marketing strategies. With increasing global awareness of environmental issues and the push towards greener technologies, promoting the eco‐friendly aspects of nanomaterials will become a critical component of marketing efforts. Highlighting the reduced environmental footprint, energy efficiency, and lower waste associated with nanomaterial‐based sensors will resonate with environmentally conscious consumers and businesses. Marketing strategies will need to incorporate messaging that aligns with sustainability trends and demonstrates how nanomaterials contribute to a greener future. Strategic collaborations and partnerships will be essential in navigating the future landscape of marketing nanomaterials. Collaborating with academic institutions, research organizations, and industry leaders can provide valuable endorsements and validate the technology's benefits. These alliances can also help in addressing regulatory challenges and expanding market reach. Joint ventures and strategic partnerships will be instrumental in accelerating market entry and leveraging combined expertise and resources to drive innovation and adoption. As regulations surrounding nanomaterials become increasingly complex, marketing strategies must address regulatory compliance and communicate transparency effectively. Marketers will need to provide clear and accurate information about how nanomaterial‐based sensors meet safety standards and regulatory requirements. This transparency will build trust with potential customers and stakeholders, mitigating concerns about the safety and efficacy of the technology. The rise of the Internet of Things (IoT) and smart technologies will open new avenues for integrating nanomaterial‐based sensors into interconnected systems. Marketing strategies will focus on demonstrating how these sensors contribute to smarter, more efficient, and automated solutions across various industries. By showcasing the role of nanomaterial sensors in enhancing IoT applications, marketers can tap into the growing demand for connected and intelligent systems. In conclusion, the future of marketing strategies for nanomaterials in sensor applications will be characterized by a blend of technological innovation, targeted digital marketing, a strong emphasis on sustainability, strategic collaborations, and regulatory transparency. By embracing these trends and leveraging new tools and approaches, companies can effectively position their nanomaterial‐based sensors, drive adoption, and capitalize on emerging opportunities in the market.

## 8 Conclusion

The commercialization of nanomaterials for sensor applications presents significant opportunities but also considerable challenges. The creation of comprehensive marketing strategies that take into account both the technical and commercial elements of product development is crucial for effectively bridging the gap between laboratory research and market acceptance. Important strategies include a deep understanding of market demands and customer needs, effective communication of the unique value propositions of nanomaterial‐based sensors, and proactive engagement with regulatory bodies to ensure compliance and facilitate market entry. Also, fostering strategic partnerships with industry stakeholders, investing in intellectual property to protect innovations, and focusing on customer education are critical for building trust and credibility in the market. The future scope of marketing strategies in nanomaterials for sensor applications is poised to undergo a profound transformation, driven by advancements in technology, evolving market demands, and increasing emphasis on sustainability.

## Conflict of Interest

The authors declare no conflict of interest.

## Author Contributions

**Anoop Singh**: Conceptualization, Methodology, Analysis, Writing. **Eliyash Ahmed**: Writing – review & editing.**Mehraj ud Din Rather**: Writing – review & editing. **Atchaya Sundararajan**: Writing – review & editing. **Alka Sharma**: Writing – review & editing. **Farah S. Choudhary**: Writing – review & editing. **Ashok K. Sundramoorthy**: Writing – review & editing. **Saurav Dixit**: Writing – review & editing. **Nikolai Ivanovich Vatin**: Writing – review & editing. **Sandeep Arya**: Conceptualization, Supervision, Methodology, Writing – review & editing.

## Data Availability

The data that support the findings of this study are available from the corresponding author upon reasonable request.

## References

[1] T. W. Ebbesen , P. M. Ajayan , Nature 1992, 358, 220.

[2] A. M. Gurban , D. Burtan , L. Rotariu , C. Bala , Sens. Actuators, B 2015, 210, 220.

[3] J. H. Lee , Sens. Actuators, B 2009, 140, 319.

[4] H. Nanto , T. Morita , H. Habara , K. Kondo , Y. Douguchi , T. Minami , Sens. Actuators, B 1996, 36, 38S4.

[5] T. Shukla , J. Sens. Technol. 2012, 2, 102.

[6] R. Savu , M. A. Ponce , E. Joanni , P. R. Bueno , M. Castro , M. Cilense , J. A. Varela , E. Longo , Mater. Res. 2009, 12, 83.

[7] M. R. Vaezi , S. K. Sadrnezhaad , Mater. Sci. Eng., B 2007, 140, 73.

[8] J. G. Lu , P. Chang , Z. Fan , Mater. Sci. Eng.: R: Rep. 2006, 52, 49.

[9] N. Joudeh , D. Linke , J. Nanobiotechnology 2022, 20, 262. 35672712 10.1186/s12951-022-01477-8 PMC9171489

[10] A. Kolmakov , M. Moskovits , Annu. Rev. Mater. Res. 2004, 34, 151.

[11] I. S. Hwang , S. J. Kim , J. K. Choi , J. Choi , H. Ji , G. T. Kim , G. Cao , J. H. Lee , Sens. Actuators, B 2010, 148, 595.

[12] X. Song , Z. Wang , Y. Liu , C. Wang , L. Li , Nanotechnology 2009, 20, 075501. 19417420 10.1088/0957-4484/20/7/075501 PMC2760478

[13] E. Sennik , N. Kilinc , Z. Z. Ozturk , J. Alloys Compd. 2014, 616, 89.

[14] S. K. Lim , S. H. Hwang , D. Chang , S. Kim , Sens. Actuators, B 2010, 149, 28.

[15] B. Geng , F. Zhan , C. Fang , N. Yu , J. Mater. Chem. 2008, 18, 4977.

[16] T. Gao , T. H. Wang , Appl. Phys. A 2005, 80, 1451.

[17] C. Baratto , G. Sberveglieri , A. Onischuk , B. Caruso , S. Di Stasio , Sens. Actuators, B 2004, 100, 261.

[18] K. Suri , S. Annapoorni , A. K. Sarkar , R. P. Tandon , Sens. Actuators, B 2002, 81, 277.

[19] Y. X. Zhang , G. H. Li , Y. X. Jin , Y. Zhang , J. Zhang , L. D. Zhang , Chem. Phys. Lett. 2002, 365, 300.

[20] S. H. Keshmiri , M. R. Rokn‐Abadi , Thin Solid Films 2001, 382, 230.

[21] V. R. Shinde , T. P. Gujar , C. D. Lokhande , R. S. Mane , S. H. Han , Mater. Sci. Eng., B 2007, 137, 119.

[22] K. Yu , Y. Zhang , R. Xu , D. Jiang , L. Luo , Q. Li , Z. Zhu , W. Lu , Solid State Commun. 2005, 133, 43.

[23] J. X. Wang , X. W. Sun , Y. Yang , H. Huang , Y. C. Lee , O. K. Tan , L. Vayssieres , Nanotechnology 2006, 17, 4995.

[24] X. Gou , R. Li , G. Wang , Z. Chen , D. Wexler , Nanotechnology 2009, 20, 495501. 19893148 10.1088/0957-4484/20/49/495501

[25] G. Wang , X. Gou , J. Horvat , J. Park , J. Phys. Chem. C 2008, 112.

[26] Report by Grand View Research , Nanomaterials Market Size, Share & Trends Analysis Report By Material (Gold, Silver, Iron, Copper), By Application (Aerospace, Automotive, Medical), By Region, And Segment Forecasts, 2024–2030.

[27] D. Jariwala , V. K. Sangwan , L. J. Lauhon , T. J. Marks , M. C. Hersam , Chem. Soc. Rev. 2013, 42, 2824. 23124307 10.1039/c2cs35335k

[28] A. Kaniyoor , R. I. Jafri , T. Arockiadoss , S. Ramaprabhu , Nanoscale 2009, 1, 382. 20648277 10.1039/b9nr00015a

[29] X. Wang , Q. Li , J. Xie , Z. Jin , J. Wang , Y. Li , K. Jiang , S. Fan , Nano Lett. 2009, 9, 3137. 19650638 10.1021/nl901260b

[30] M. M. Barsan , M. E. Ghica , C. M. Brett , Anal. Chim. Acta 2015, 881, 1. 26041516 10.1016/j.aca.2015.02.059

[31] Y. Liao , Q. Li , N. Wang , S. Shao , Sens. Actuators, B 2015, 215, 592.

[32] K. Chen , W. Gao , S. Emaminejad , D. Kiriya , H. Ota , H. Y. Y. Nyein , K. Takei , A. Javey , Adv. Mater. 2016, 28, 4397. 26880046 10.1002/adma.201504958

[33] J. J. Gooding , Electrochim. Acta 2005, 50, 3049.

[34] K. Y. Chun , Y. Oh , J. Rho , J. H. Ahn , Y. J. Kim , H. R. Choi , S. Baik , Nat. Nanotechnol. 2010, 5, 853. 21113161 10.1038/nnano.2010.232

[35] G. Bhanjana , N. Dilbaghi , K. H. Kim , S. Kumar , J. Mol. Liq. 2017, 244, 506.

[36] X. Luo , J. Zeng , S. Liu , L. Zhang , Bioresour. Technol. 2015, 194, 403. 26216781 10.1016/j.biortech.2015.07.044

[37] Z. Zhang , Z. Wang , Q. Li , H. Zou , Y. Shi , Talanta 2014, 119, 613. 24401463 10.1016/j.talanta.2013.11.010

[38] Y. Li , G. Peng , Q. He , H. Zhu , S. M. Al‐Hamadani , Spectrochim. Acta, Part A 2015, 140, 156. 10.1016/j.saa.2014.12.091 25590827

[39] R. Sitko , P. Janik , B. Zawisza , E. Talik , E. Margui , I. Queralt , Anal. Chem. 2015, 87, 3535. 25707847 10.1021/acs.analchem.5b00283

[40] G. Bhanjana , N. Dilbaghi , S. Chaudhary , K. H. Kim , S. Kumar , Analyst 2016, 141, 4211. 27141553 10.1039/c5an02663f

[41] B. Cheng , L. Zhou , L. Lu , J. Liu , X. Dong , F. Xi , P. Chen , Sens. Actuators, B 2018, 259, 364.

[42] A. Simpson , R. R. Pandey , C. C. Chusuei , K. Ghosh , R. Patel , A. K. Wanekaya , Carbon 2018, 127, 122.

[43] A. K. Wanekaya , W. Chen , A. Mulchandani , J. Environ. Monitoring 2008, 10. 10.1039/b806830p 18528536

[44] E. N. Primo , F. A. Gutierrez , M. D. Rubianes , G. A. Rivas , Electrochim. Acta 2015, 182, 391.

[45] S. H. Wen , Y. Wang , Y. H. Yuan , R. P. Liang , J. D. Qiu , Anal. Chim. Acta 2018, 1002, 82. 29306416 10.1016/j.aca.2017.11.057

[46] P. Wahed , M. A. Razzaq , S. Dharmapuri , M. Corrales , Food Chem. 2016, 202, 476. 26920321 10.1016/j.foodchem.2016.01.136

[47] B. Hoffmann , S. Münch , F. Schwägele , C. Neusüß , W. Jira , Food Control 2017, 71, 200.

[48] M. Jiménez‐Salcedo , M. T. Tena , J. Chromatogr. A 2017, 1487, 14. 28129937 10.1016/j.chroma.2017.01.042

[49] M. M. A. Omar , A. A. Elbashir , O. J. Schmitz , Food Chem. 2017, 214, 300. 27507479 10.1016/j.foodchem.2016.07.060

[50] L. Rotariu , F. Lagarde , N. Jaffrezic‐Renault , C. Bala , TrAC, Trends Anal. Chem. 2016, 79, 80.

[51] G. Herzog , V. Kam , A. Berduque , D. W. Arrigan , J. Agric. Food Chem. 2008, 56, 4304. 18512937 10.1021/jf7035966

[52] M. Elyasi , M. A. Khalilzadeh , H. Karimi‐Maleh , Food Chem. 2013, 141, 4311. 23993620 10.1016/j.foodchem.2013.07.020

[53] J. Sun , Y. Lu , L. He , J. Pang , F. Yang , Y. Liu , Trends Anal. Chem. 2019, 122, 115754.

[54] V. Montes‐Garcia , M. A. Squillaci , M. Diez‐Castellnou , Q. K. Ong , F. Stellacci , P. Samori , Chem. Soc. Rev. 2021, 50, 1269. 33290474 10.1039/d0cs01112f

[55] A. C. M. Hacke , D. Lima , S. Kuss , J. Electroanal. Chem. 2022, 922, 116786.

[56] M. Forough , K. Farhadi , Turk. J. Eng. Environ. Sci. 2010, 34, 281.

[57] K. Farhadi , M. Forough , R. Molaei , S. Hajizadeh , A. Rafipour , Sens. Actuators, B 2012, 161, 880.

[58] T. N. J. I. Edison , R. Atchudan , Y. R Lee , J. Clust. Sci. 2016, 27, 683.

[59] S. Pandey , G. K. Goswami , K. K. Nanda , Int. J. Biol. Macromol. 2012, 51, 583. 22750580 10.1016/j.ijbiomac.2012.06.033

[60] N. Srikhao , P. Kasemsiri , N. Lorwanishpaisarn , M. Okhawilai , Res. Chem. Intermed. 2021, 47, 1269.

[61] K. R. Aadila , N. Pandey , S. I. Mussatto , H. Jha , J. Environ. Chem. Eng. 2019, 7, 103296.

[62] M. R. Bindhu , M. Umadevi , G. A. Esmail , N. A. Al‐Dhabi , M. V Arasu , J. Photochem. Photobiol. B Biol. 2020, 205, 111836. 10.1016/j.jphotobiol.2020.111836 32172135

[63] A. Aravind , M. Sebastian , B. Mathew , New J. Chem. 2018, 42, 15022.

[64] A. Aravind , M. Sebastian , B. Mathew , Environ. Sci. Water Res. Technol. 2018, 4, 1531.

[65] E. Turunc , O. Kahraman , R. Binzet , Anal. Biochem. 2021, 621, 114123. 33549546 10.1016/j.ab.2021.114123

[66] F. Zamarchi , I. C Vieira , J. Pharm. Biomed. Anal. 2021, 196, 113912. 33581590 10.1016/j.jpba.2021.113912

[67] R. Teimuri‐Mofrad , R. Hadi , B. Tahmasebi , S. Farhoudian , M. Mehravar , R. Nasiri , Nanochem. Res. 2017, 2, 8.

[68] K. X. Lee , K. Shameli , Y. P. Yew , S. Y. Teow , H. Jahangirian , R. Rafiee‐Moghaddam , T. J. Webster , Int. J. Nanomed. 2020, 15, 275. 10.2147/IJN.S233789 PMC6970630 32021180

[69] S. Pandey , G. K. Goswami , K. K. Nanda , Carbohydr. Polym. 2013, 94, 229. 23544533 10.1016/j.carbpol.2013.01.009

[70] B. R. Gangapuram , R. Bandi , R. Dadigala , G. M. Kotu , V. Guttena , J. Clust. Sci. 2017, 28, 2873.

[71] M. Bahram , E. Mohammadzadeh , Anal. Methods 2014, 6, 6916.

[72] Z. Ashikbayeva , A. Bekmurzayeva , Z. Myrkhiyeva , N. Assylbekova , T. S. Atabaev , D. Tosi , Opt. Laser Technol. 2023, 161, 109136.

[73] S. Nazarpour , R. Hajian , M. H Sabzvaria , Microchem. J. 2020, 154, 104634.

[74] M. A. Tabrizia , J. N Varkanib , Sens. Actuators, B 2014, 202, 475.

[75] R. Karthik , M. Govindasamy , S.‐M. Chen , V. Mani , B.‐S. Lou , R. Devasenathipathy , Y.‐S. Hou , A. Elangovan , J. Colloid Interface Sci. 2016, 475, 46. 27153217 10.1016/j.jcis.2016.04.044

[76] F. J. González‐Fuentes , G. A. Molina , R. Silva , J. L. López‐Miranda , R. Esparza , A. R. Hernandez‐Martinez , M. Estevez , Sensors 2020, 20, 6108. 33121053 10.3390/s20216108 PMC7662439

[77] A. E. Nel , L. Madler , D. Velegol , T. Xia , E. M. V. Hoek , P. Somasundaran , F. Klaessig , V. Castranova , M. Thompson , Nat. Mater. 2009, 8, 543. 19525947 10.1038/nmat2442

[78] M. A. Blesa , A. D. Weisz , P. J. Morando , J. A. Salfity , G. E. Magaz , A. E. Regazzoni , Coord. Chem. Rev. 2000, 196, 31.

[79] M. J. Limo , A. Sola‐Rabada , E. Boix , V. Thota , Z. C. Westcott , V. Puddu , C. C. Perry , Chem. Rev. 2018, 118, 11118. 30362737 10.1021/acs.chemrev.7b00660

[80] L. T. Zegebreal , N. A. Tegegne , F. G. Hone , Sens. Actuators, A 2023, 359, 114472.

[81] M. L. Pierce , C. B. Moore , Water Res. 1982, 16, 1247.

[82] B. A. Manning , S. E. Fendorf , S. Goldberg , Environ. Sci. Technol. 1998, 32, 2383.

[83] C.‐H. Liu , Y.‐H. Chuang , T.‐Y. Chen , Y. Tian , H. Li , M.‐K. Wang , W. Zhang , Environ. Sci. Technol. 2015, 49, 7726. 26055623 10.1021/acs.est.5b00381

[84] Y. Liu , K. Ai , L. Lu , Chem. Rev. 2014, 114, 5057. 24517847 10.1021/cr400407a

[85] Q. Ye , F. Zhou , W. Liu , Chem. Soc. Rev. 2011, 40, 4244. 21603689 10.1039/c1cs15026j

[86] T. Paunesku , T. Rajh , G. Wiederrecht , J. Maser , S. Vogt , N. Stojicevic , M. Protic , B. Lai , J. Oryhon , M. Thurnauer , G. Woloschak , Nat. Mater. 2003, 2, 343. 12692534 10.1038/nmat875

[87] C. Xu , K. Xu , H. Gu , R. Zheng , H. Liu , X. Zhang , Z. Guo , B. Xu , J. Am. Chem. Soc. 2004, 126, 9938. 15303865 10.1021/ja0464802

[88] M. A. Blesa , P. J. Morando , A. E. Regazzoni , Chemical Dissolution of Metal Oxides, CRC Press, Boca Raton, FL 1994.

[89] M. D. Shultz , J. U. Reveles , S. N. Khanna , E. E. Carpenter , J. Am. Chem. Soc. 2007, 129, 2482. 17290990 10.1021/ja0651963

[90] H. Fan , Z. Zhao , G. Yan , X. Zhang , C. Yang , H. Meng , Z. Chen , H. Liu , W. Tan , Angew. Chem., Int. Ed. 2015, 54, 4801. 10.1002/anie.201411417 25728966

[91] K. N. Chaudhari , N. K. Chaudhari , J.‐S. Yu , Catal. Sci. Technol. 2012, 2, 124.

[92] R. André , F. Natálio , M. Humanes , J. Leppin , K. Heinze , R. Wever , H.‐C. Schröder , W. E. G. Müller , W. Tremel , Adv. Funct. Mater. 2011, 21, 501.

[93] J. Mu , Y. Wang , M. Zhao , L. Zhang , Chem. Commun. 2012, 48, 2540. 10.1039/c2cc17013b 22288077

[94] J. Dong , L. Song , J.‐J. Yin , W. He , Y. Wu , N. Gu , Y. Zhang , ACS Appl. Mater. Interfaces 2014, 6, 1959. 24387092 10.1021/am405009f

[95] W. Chen , J. Chen , A.‐L. Liu , L.‐M. Wang , G.‐W. Li , X.‐H. Lin , ChemCatChem 2011, 3, 1151.

[96] A. Asati , S. Santra , C. Kaittanis , S. Nath , J. M. Perez , Angew. Chem., Int. Ed. 2009, 121, 2348. 10.1002/anie.200805279 PMC2923475 19130532

[97] D. Li , B. Liu , P.‐J. J. Huang , Z. Zhang , J. Liu , Chem. Commun. 2018, 54, 12519. 10.1039/c8cc07062h 30345454

[98] T. Pirmohamed , J. M. Dowding , S. Singh , B. Wasserman , E. Heckert , A. S. Karakoti , J. E. S. King , S. Seal , Chem. Commun. 2010, 46, 2736. 10.1039/b922024k PMC3038687 20369166

[99] C. Korsvik , S. Patil , S. Seal , W. T. Self , Chem. Commun. 2007 1056. 10.1039/b615134e 17325804

[100] M. H. Kuchma , C. B. Komanski , J. Colon , A. Teblum , A. E. Masunov , B. Alvarado , S. Babu , S. Seal , J. Summy , C. H. Baker , Nanomedicine: NBM 2010, 6, 738. 10.1016/j.nano.2010.05.004 20553964

[101] Y. Huang , J. Ren , X. Qu , Chem. Rev. 2019, 119, 4357. 30801188 10.1021/acs.chemrev.8b00672

[102] J. Wu , X. Wang , Q. Wang , Z. Lou , S. Li , Y. Zhu , L. Qin , H. Wei , Chem. Soc. Rev. 2019, 48, 1004. 30534770 10.1039/c8cs00457a

[103] U. O. Aigbe , O. A. Osibote , J. Hazard. Mater. Adv. 2024, 13, 100401.

[104] A. Shanmugavel , E. R. Rene , S. P. Balakrishnan , N. Krishnakumar , S. P. Jose , Environ. Res. 2024, 119544. 38969312 10.1016/j.envres.2024.119544

[105] V. G. Reshma , P. V. Mohanan , J. Lumin. 2019, 205, 287.

[106] K. Sreeja , Doctoral dissertation , Sree Neelakanta Government Sanskrit College, Pattambi, 2022.

[107] T. A. Tabish , H. Hayat , A. Abbas , R. J. Narayan , Curr. Opin. Electrochem. 2021, 30, 100786.

[108] R. Banerjee , P. Goswami , M. Chakrabarti , D. Chakraborty , A. Mukherjee , A. Mukherjee , Mut. Res./Genetic Toxicol. Environ. Mutagenesis 2021, 865, 503338. 10.1016/j.mrgentox.2021.503338 33865544

[109] H. Yoon , Nanomaterials 2013, 3, 524. 28348348 10.3390/nano3030524 PMC5304658

[110] D. W. Hatchett , M. Josowicz , Chem. Rev. 2008, 108, 746. 18171087 10.1021/cr068112h

[111] S. K. Metkar , K. Girigoswami , Biocatalysis Agric. Biotechnol. 2019, 17, 271.

[112] A. Verma , R. Gupta , A. S. Verma , T. Kumar , Sens. Actuators Rep. 2023, 5, 100143.

[113] S. Wu , P. Liu , Y. Zhang , H. Zhang , X. Qin , Sens. Actuators, B 2017, 252, 697.

[114] M. Ramesh , R. Janani , C. Deepa , L. Rajeshkumar , Biosensors 2022, 13, 40. 36671875 10.3390/bios13010040 PMC9856107

[115] A. Bigdeli , F. Ghasemi , H. Golmohammadi , S. Abbasi‐Moayed , M. A. F. Nejad , N. Fahimi‐Kashani , S. Jafarinejad , M. Shahrajabian , M. R. Hormozi‐Nezhad , Nanoscale 2017, 9, 16546. 29083011 10.1039/c7nr03311g

[116] V. C. Gungor , G. P. Hancke , IEEE Trans. Ind. Electron. 2009, 56.

[117] J. Burgués , J. M. Jiménez‐Soto , S. Marco , Anal. Chim. Acta 2018, 1013, 13. 29501088 10.1016/j.aca.2018.01.062

[118] M. Farré , L. Kantiani , M. Petrovic , S. Pérez , D. Barceló , J. Chromatogr. A 2012, 1259, 86. 22877973 10.1016/j.chroma.2012.07.024

[119] L. A. Currie , Anal. Chim. Acta 1999, 391, 127.

[120] P. Yang , J. Pang , F. Hu , J. Peng , D. Jiang , Z. Chu , W. Jin , Sens. Actuator B Chem. 2018, 276, 31.

[121] J. Yoon , S. N. Lee , M. K. Shin , H.‐W. Kim , H. K. Choi , T. Lee , J.‐W. Choi , Biosens. Bioelectron. 2019, 140, 111343. 31150985 10.1016/j.bios.2019.111343

[122] H. Rabie , Y. Zhang , N. Pasquale , M. J. Lagos , P. E. Batson , K. N. I. R. Lee , Adv. Mater. 2019, 31, 1806991. 10.1002/adma.201806991 PMC8849937 30761616

[123] H. He , B. Liu , S. Wen , J. Liao , G. Lin , J. Zhou , D. Jin , Anal. Chem. 2018, 90, 12356. 30335361 10.1021/acs.analchem.8b04330

[124] S. Kumar , Y. Lei , N. H. Alshareef , M. A. Quevedo‐Lopez , K. N. Salama , Biosens. Bioelectron. 2018, 121, 243. 30219724 10.1016/j.bios.2018.08.076

[125] P. Zhang , S. Yang , R. Pineda‐Gómez , B. Ibarlucea , J. Ma , M. R. Lohe , T. F. Akbar , L. Baraban , G. Cuniberti , X. Feng , Small 2019, 15, 1901265. 10.1002/smll.201901265 31034144

[126] A. S. Ghrera , C. M. Pandey , B. D Malhotra , Sens. Actuator B Chem. 2018, 266, 329.

[127] L. Lu , L. Zhou , J. Chen , F. Yan , J. Liu , X. Dong , F. Xi , P. Chen , ACS Nano 2018, 12, 12673. 30485066 10.1021/acsnano.8b07564

[128] T. García‐Mendiola , I. Bravo , J. M. López‐Moreno , F. Pariente , R. Wannemacher , K. Weber , J. Popp , E. Lorenzo , Sens. Actuator B Chem. 2018, 256, 233.

[129] C. Y. Lee , L. P. Wu , T.‐T. Chou , Y.‐Z. Hsieh , Sens. Actuator B Chem. 2018, 257, 672.

[130] C. N. Loynachan , M. R. Thomas , E. R. Gray , D. A. Richards , J. Kim , B. S. Miller , J. C. Brookes , S. Agarwal , V. Chudasama , R. A. McKendry , M. M. Stevens , ACS Nano 2018, 12, 279. 29215864 10.1021/acsnano.7b06229 PMC5785759

[131] Z. Cheng , N. Choi , R. Wang , S. Lee , K. C. Moon , S.‐Y. Yoon , L. Chen , J. Choo , ACS Nano 2017, 11, 4926. 28441008 10.1021/acsnano.7b01536

[132] B. P. Wong , B. Kerkez , Environ. Model. Software 2016, 84, 505.

[133] P. Mathiyalagan , S. Pokhriyal , Improvements in Industrial Sensors: Increasing Reliability and Efficiency 2024, Available at SSRN 5083750.

[134] N. Musa , N. B. Singh , Advanced Sensors for Smart Healthcare, Elsevier, Amsterdam, The Netherlands 2025, pp. 47–67.

[135] V. Chaudhary , P. Gaur , S. Rustagi , Sustainable Mater. Technol. 2024, e00952.

[136] M. Talbi , A. Al‐Hamry , P. R. Teixeira , L. G. Paterno , M. B. Ali , O. Kanoun , Chemosensors 2022, 10, 40.

[137] K. Chan , D. N. Schillereff , A. C. Baas , M. A. Chadwick , B. Main , M. Mulligan , F. T. O'Shea , R. Pearce , T. E. Smith , A. Van Soesbergen , E. Tebbs , Progr. Phys. Geogr.: Earth Environ. 2021, 45, 305.

[138] D. Li , Y. Wang , J. Wang , C. Wang , Y. Duan , Sens. Actuators, A 2020, 309, 111990.

[139] B. Singh , A. Singh , A. Sharma , P. Mahajan , S. Verma , B. Padha , A. Ahmed , S. Arya , J. Mol. Struct. 2022, 1255, 132379.

[140] A. Swain , E. Abdellatif , A. Mousa , P. W. Pong , Energies 2022, 15, 7339.

[141] B. Miranda , I. Rea , P. Dardano , L. De Stefano , C. Forestiere , Biosensors 2021, 11, 107. 33916580 10.3390/bios11040107 PMC8066870

[142] F. Wang , S. Cao , R. Yan , Z. Wang , D. Wang , H. Yang , Sensors 2017, 17, 2689. 29160798 10.3390/s17112689 PMC5713634

[143] R. K. Joshi , J. J. Schneider , Chem. Soc. Rev. 2012, 41, 5285.22722888

[144] M. I. Dafinone , G. Feng , T. Brugarolas , K. E. Tettey , D. Lee , ACS Nano 2011, 5. 10.1021/nn201167j 21557541

[145] K. Kant , R. Beeram , L. G. Cabaleiro , Y. Cao , D. Quesada‐González , H. Guo , S. Gomez‐Grana , Y. Joung , S. Kothadiya , D. García‐Lojo , M. Lafuente , Nanoscale Horiz. 2024. 10.1039/d4nh00226a PMC11378978 39240539

[146] K. A. Olu‐lawal , O. K. Olajiga , E. C. Ani , A. K. Adeleke , D. J. P. Montero , Eng. Sci. Technol. J. 2024, 5, 728.

[147] T. Yang , D. Xie , Z. Li , H. Zhu , Mater. Sci. Eng.: R: Rep. 2017, 115, 1.

[148] M. Yazdi , Advances in Computational Mathematics for Industrial System Reliability and Maintainability, Springer Nature Switzerland, Cham 2024, pp. 79–103.

[149] M. Colledani , T. Tolio , A. Fischer , B. Iung , G. Lanza , R. Schmitt , J. Váncza , CIRP Ann. 2014, 63, 773.

[150] C. Dincer , R. Bruch , E. Costa‐Rama , M. T. Fernández‐Abedul , A. Merkoçi , A. Manz , G. A. Urban , F. Güder , Adv. Mater. 2019, 31, 1806739. 10.1002/adma.201806739 31094032

[151] Y. Fu , T. Liu , H. Wang , Z. Wang , L. Hou , J. Jiang , T. Xu , J. Sci.: Adv. Mater. Devices 2024, 100694.

[152] J. W. Hui , D. E. Culler , IEEE Internet Computing 2008, 12.

[153] M. K. Vanteru , K. A. Jayabalaji , P. Ilango , B. Nautiyal , A. Y. Begum , Measurement: Sensors 2023, 28, 100826.

[154] S. Diez , S. Lacy , H. Coe , J. Urquiza , M. Priestman , M. Flynn , N. Marsden , N. A. Martin , S. Gillott , T. Bannan , P. M. Edwards , Atmospher. Measur. Techn. 2024, 17, 3809.

[155] M. S. Soares , R. Singh , S. Kumar , R. Jha , J. Nedoma , R. Martinek , C. Marques , Opt. Laser Technol. 2024, 177, 111049.

[156] G. Breglio , R. Bernini , G. M. Berruti , F. A. Bruno , S. Buontempo , S. Campopiano , E. Catalano , M. Consales , A. Coscetta , A. Cutolo , M. A. Cutolo , Sensors 2023, 23, 3187.36991894

[157] C. Cacciuttolo , V. Guzmán , P. Catriñir , E. Atencio , S. Komarizadehasl , J. A. Lozano‐Galant , Sensors 2023, 23, 6846. 37571628 10.3390/s23156846 PMC10422650

[158] A. Soussi , E. Zero , R. Sacile , D. Trinchero , M. Fossa , Sensors 2024, 24, 2647. 38676264 10.3390/s24082647 PMC11053448

[159] M. AtaeiKachouei , A. Kaushik , M. A. Ali , Advanced Intelligent Systems 2023, 5, 2300321.

[160] H. Zhu , X. Huang , Y. Deng , H. Chen , M. Fan , Z. Gong , TrAC, Trends Anal. Chem. 2023, 158, 116879.

[161] M. Maruthupandi , D. Thiruppathi , N. Vasimalai , J. Hazard. Mater. 2020, 392, 122294. 32105954 10.1016/j.jhazmat.2020.122294

[162] N. Vasimalai , M. T. Fernández‐Argüelles , B. Espiña , ACS Appl. Mater. Interfaces 2018, 10, 1634. 29271189 10.1021/acsami.7b11769

[163] A. Kumar , M. Castro , J. F. Feller , Sensors 2023,, 23, 4017. 37112358 10.3390/s23084017 PMC10141392

[164] P. Overbosch , S. Blanchard , in Food safety management (Eds: V. Andersen , H. Lelieveld , Y. Motarjemi ), Academic Press, Cambridge, MA 2023, pp. 497–512.

[165] C. M. Fernandez , J. Alves , P. D. Gaspar , T. M. Lima , P. D. Silva , J. Sci. Food Agric. 2023, 103, 986. 35279845 10.1002/jsfa.11863

[166] A. Hassoun , N. Alhaj Abdullah , A. Aït‐Kaddour , M. Ghellam , A. Beşir , O. Zannou , B. Onal , R. M. Aadil , J. M. Lorenzo , A. Mousavi Khaneghah , J. M. Regenstein , Crit. Rev. Food Sci. Nutr. 2024, 64, 873. 35950635 10.1080/10408398.2022.2110033

[167] R. Abedi‐Firoozjah , H. Ebdali , M. Soltani , P. Abdolahi‐Fard , M. Heydari , E. Assadpour , M. Azizi‐Lalabadi , F. Zhang , S. M. Jafari , Coord. Chem. Rev. 2024, 500, 215545.

[168] A. Thakur , A. Kumar , Sci. Total Environ. 2022, 834, 155219. 35421493 10.1016/j.scitotenv.2022.155219

[169] S. B. Sukhavasi , S. B. Sukhavasi , K. Elleithy , S. Abuzneid , A. Elleithy , Sensors 2021, 21, 488.33445557

[170] W. Q. Wang , D. Jiang , IEEE Sens. J. 2014, 14.

[171] A. Munir , A. Aved , E. Blasch , AI 2022, 3.

[172] M. Shimoni , R. Haelterman , C. Perneel , IEEE Geosci. Remote Sensing Mag. 2019, 7.

[173] P. Fraga‐Lamas , T. M. Fernández‐Caramés , M. Suárez‐Albela , L. Castedo , M. González‐López , Sensors 2016, 16, 1644. 27782052 10.3390/s16101644 PMC5087432

[174] E. Blasch , T. Pham , C. Y. Chong , W. Koch , H. Leung , D. Braines , T. Abdelzaher , IEEE Aerospace Electron. Syst. Mag. 2021, 36.

[175] O. Gohardani , M. C. Elola , C. Elizetxea , Progr. Aerospace Sci. 2014, 70, 42.

[176] T. Ahmad , D. Zhang , Sustain. Cities Soc. 2021, 68, 102783. 10.1016/j.scs.2021.102784 PMC7894099 33643810

[177] T. Ahmad , D. Zhang , C. Huang , H. Zhang , N. Dai , Y. Song , H. Chen , J. Cleaner Prod. 2021, 289, 125834.

[178] M. Ali , K. Prakash , M. A. Hossain , H. R. Pota , J. Cleaner Prod. 2021, 314, 127904.

[179] A. E. L. Rivas , T. Abrao , Electr. Power Syst. Res. 2020, 189, 106602.

[180] S. A. A. Abir , A. Anwar , J. Choi , A. S. M. Kayes , IEEE Access 2021, 9, 50961.

[181] M. Alonso , H. Amaris , D. Alcala , D. M. Florez R , Sensors 2020, 20, 2187. 32294923 10.3390/s20082187 PMC7218892

[182] S. Park , S. Jayaraman , in Wearable Sensors, Academic Press, Cambridge, MA 2021, pp. 3–27.

[183] A. Jain , V. Kanhangad , IEEE Sens. J. 2017, 18.

[184] A. Allouch , A. Koubâa , T. Abbes , A. Ammar , IEEE Sens. J. 2017, 17.

[185] H. P. Gupta , H. S. Chudgar , S. Mukherjee , T. Dutta , K. Sharma , IEEE Sens. J. 2016, 16.

[186] G. K. Moinudeen , F. Ahmad , D. Kumar , Y. Al‐Douri , S. Ahmad , Int. J. Internet Things 2017, 6, 106.

[187] S. Mao , L. Fu , C. Yin , X. Liu , H. Karimi‐Maleh , RSC Adv. 2022, 12, 22592. 36105989 10.1039/d2ra04162f PMC9372877

[188] R. Nester , Nanosensor Market Size & Share, By Type, Application – Global Supply & Demand Analysis, Growth Forecasts, Statistics Report 2024–2036. [Online] Nanosensors Market, 2024, https://www.researchnester.com/reports/nanosensors‐market/3083 (accessed: September 2024).

[189] Global Market Insights , By Technology (Molecular Self Assembly, Top‐down Assembly, Bottom‐up Assembly), By End User & Forecast, 2024–2032 , [Online] Global Market Insights, 2024, Available at: https://www.gminsights.com/industry‐analysis/nanosensor‐market (accessed: September 2024).

[190] S. Gottardo , A. Mech , J. Drbohlavová , A. Małyska , S. Bøwadt , J. R. Sintes , H. Rauscher , NanoImpact 2021, 21, 100297. 33738354 10.1016/j.impact.2021.100297 PMC7941606

[191] C. A. Charitidis , P. Georgiou , M. A. Koklioti , A. F. Trompeta , V. Markakis , Manuf. Rev. 2014, 1, 11.

[192] M. Ateia , H. Wei , S. Andreescu , Environ. Sci. Technol. 2024, 58, 2636. 38302436 10.1021/acs.est.3c09889

[193] J. G. Siri , C. A. Fernando , S. N. De Silva , Recent Pat. Nanotechnol. 2020, 14, 307. 32532198 10.2174/1872210514666200612174317

[194] Y. Luo , M. R. Abidian , J. H. Ahn , D. Akinwande , A. M. Andrews , M. Antonietti , Z. Bao , M. Berggren , C. A. Berkey , C. J. Bettinger , J. Chen , ACS Nano 2023, 17, 5211. 36892156 10.1021/acsnano.2c12606 PMC11223676

[195] H. M. Saleh , A. I. Hassan , Sustainability 2023, 15, 10891.

[196] J. Z. Raja , T. Frandsen , C. Kowalkowski , M. Jarmatz , J. Business Res. 2020, 114, 142.

